# Dynamic Response of *Pseudomonas putida* S12 to Sudden Addition of Toluene and the Potential Role of the Solvent Tolerance Gene *trgI*

**DOI:** 10.1371/journal.pone.0132416

**Published:** 2015-07-16

**Authors:** Rita J. M. Volkers, L. Basten Snoek, Harald J. Ruijssenaars, Johannes H. de Winde

**Affiliations:** 1 Laboratory of Nematology, Plant Sciences, Wageningen University, Wageningen, The Netherlands; 2 Corbion, Gorinchem, The Netherlands; 3 Department of Biotechnology,Faculty of Applied Sciences, Delft University of Technology, Delft, The Netherlands; 4 Kluyver Centre for Genomics of Industrial Fermentation, Delft, The Netherlands; Louisiana State University and A & M College, UNITED STATES

## Abstract

*Pseudomonas putida* S12 is exceptionally tolerant to various organic solvents. To obtain further insight into this bacterium’s primary defence mechanisms towards these potentially harmful substances, we studied its genome wide transcriptional response to sudden addition of toluene. Global gene expression profiles were monitored for 30 minutes after toluene addition. During toluene exposure, high oxygen-affinity cytochrome *c* oxidase is specifically expressed to provide for an adequate proton gradient supporting solvent efflux mechanisms. Concomitantly, the glyoxylate bypass route was up-regulated, to repair an apparent toluene stress-induced redox imbalance. A knock-out mutant of *trgI*, a recently identified toluene-repressed gene, was investigated in order to identify TrgI function. Remarkably, upon addition of toluene the number of differentially expressed genes initially was much lower in the *trgI*-mutant than in the wild-type strain. This suggested that after deletion of *trgI* cells were better prepared for sudden organic solvent stress. Before, as well as after, addition of toluene many genes of highly diverse functions were differentially expressed in *trgI*-mutant cells as compared to wild-type cells. This led to the hypothesis that TrgI may not only be involved in the modulation of solvent-elicited responses but in addition may affect basal expression levels of large groups of genes.

## Introduction

*Pseudomonas putida* S12 is an exceptionally solvent-tolerant bacterium that thrives in the presence of organic solvents such as 1-octanol, toluene and benzene and as such is important for the production of industrially relevant chemicals [1–4]. Several mechanisms of solvent tolerance have been identified in this organism, the most important of which is the solvent extrusion pump SrpABC that is located in the cytoplasmic membrane. This RND-type transporter actively removes solvent molecules from the membrane at the expense of the proton gradient [5,6].

Previously, we reported on the proteome and transcriptome responses of *P*. *putida* S12 to toluene [7,8]. Several of the observed responses confirmed previous findings, *e*.*g*., the induction of the solvent extrusion pump SrpABC and differential expression of membrane-associated and stress-response genes. A novel finding was the differential expression of energy-management systems. This response may compensate for the dissipation of the proton gradient associated with solvent stress, since the accumulation of solvent molecules in the cytoplasmic membrane brings about increased permeability for protons (uncoupling effect). In addition, SrpABC-mediated solvent extrusion draws on the proton gradient. Moreover, a novel gene involved in solvent tolerance, *trgI*, was identified. The expression of *trgI* decreased immediately and very rapidly after sudden addition of toluene. In steady-state chemostats, i.e, under fully toluene-adapted conditions, expression of both the gene and its encoded protein was significantly down-regulated in the presence of 5 mM toluene [7,8]. Deletion of *trgI* [7] resulted in a more rounded cell morphology and increased resistance to sudden toluene shock. Although the precise function of *trgI* remained obscure, we hypothesise that it is involved in the first line of defence against solvents [7].

The immediate down-regulation of *trgI* upon toluene exposure provoked the question how expression of other genes in *P*. *putida* S12 would respond to the sudden addition of toluene. This would provide valuable insights into the cellular functions involved in the early adaptation response to organic solvents. Although previously several-omics studies of solvent-exposed microorganisms have been published (for example [7–12]), none of these involved a genome-wide monitoring of the early adaptation response. Instead, batch cultures were sampled at a single time-point, or steady-state chemostat cultures that were fully adapted to organic solvent were analysed. Therefore, we chose to study the global gene expression profiles of *P*. *putida* S12 during the first 30 minutes after the addition of toluene. In addition to wild type *P*. *putida* S12, the *trgI* knock-out mutant *P*. *putida* S12Δ*trgI* was investigated to shed more light onto the role of this gene in the early solvent tolerance response.

## Materials and Methods

### Bacterial strains

The bacterial strains used in this study are *Pseudomonas putida* S12, which was originally isolated as a styrene utilising bacterium [13] and *P*. *putida* S12∆*trgI*. *P*. *putida* S12∆*trgI* is a *trgI* knock-out mutant that was constructed as described previously [7].

### Standard culture conditions

Luria-Bertani broth (LB medium) [14] was used as the standard culturing medium. As a solid medium, LB with 1.5% (w/v) agar was used. Batch cultivation was routinely carried out in 100-ml Erlenmeyer flasks containing 25 ml of liquid medium, placed on a horizontally shaking incubator at 30°C.

### Analysis of differential gene expression after a sudden addition of toluene

#### Culture conditions and sampling

Differential gene expression after a sudden addition of 5 mM toluene was analysed in early exponential phase cultures (optical density at 600 nm of 0.5–0.6) of *P*. *putida* strains S12 (wild-type) and S12Δ*trgI*. The cells were grown in 100 ml of LB medium in 1-L bottles placed on a horizontally shaking incubator at 30°C. Samples of 1 ml were drawn with a syringe through the rubber-covered hole in the screw cap of the bottle immediately before (t = 0) and at set intervals (1, 2, 5, 10 and 30 minutes) after toluene addition. See [7] for details about sampling.

#### Transcriptome analysis

mRNA isolation, cDNA preparation and hybridisation for transcriptome analysis were performed as described previously [7]. The custom made high-density microarrays used were based on the genome sequence of *P*. *putida* S12 (GenBank accession AYKV00000000.1). The microarray results, as well as the microarray itself, were made public in Gene Expression Omnibus, accession GSE64791.

#### Data analysis

Microarray data were imported into the GeneSpring GX 7.3.1 software package (Agilent Technologies, Santa Clara, CA, USA) using the GC RMA algorithm. After normalisation (signals below 0.01 were taken as 0.01; per chip: normalise to 50^th^ percentile; per gene: normalise to median) of the data, probe sets representing control genes were removed, as well as absolute non-changing loci (between 0.667- and 1.334-fold change). The resulting set of 6164 differentially expressed loci was used for further investigation.

**Fig 1:**
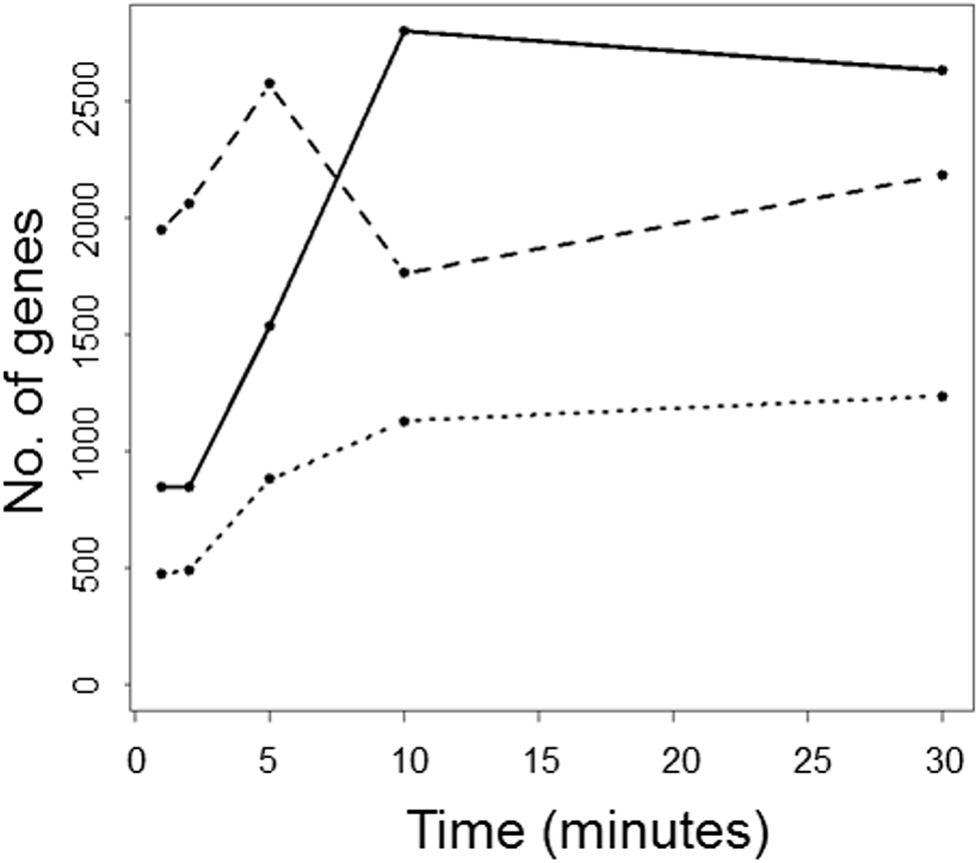
Summary of the transcriptomics results. Number of genes differentially expressed with an absolute log2(expression level at t = 0 / expression level at t = t) ≥0.5 in wild-type S12 (dashed line) and mutant S12Δ*trgI* (solid line) at the indicated time points are shown. The dotted line shows the number of genes that is present in both comparisons. T = 0: no toluene present.

The overall transcriptional activity change during toluene exposure was quantified by calculating the total number of differentially expressed genes for each time point after toluene addition, using the transcript levels at t = 0 as reference (Fig 1).

#### Overrepresentation and statistical analysis

Overrepresentation of specific groups of genes among the total response-groups was determined using the hyper-geometric test in the R statistical program environment. For example: in a group of genes selected for up-regulation following toluene exposure, the chance that an x-number of genes of COG group A are in that group of up-regulated genes is being assessed. Or, in other words, it can be assessed what the chance is that this group of up-regulated genes contains 50% COG group A while all genes contain 30% COG group A. The comparison between sets of two such groups (in this example ‘up-regulated’ and ‘COG group A’) can be found in Table 1.

**Table 1:** Classification of genes by effect of *trgI* deletion on intrinsic expression level and toluene responsiveness.

Group	Effect of *trgI* deletion	No. of genes (% of total)	Overrepresented COGs^c^
1	None	1287 (21%)	OHJFD
2	Intrinsic expression level^a^ altered; no effect on toluene responsiveness^b^	472 (8%)	CO
3	Toluene responsiveness^b^ gained	1762 (29%) (473 genes with altered intrinsic expression level^a^)	JUEG
4	Toluene responsiveness^b^ lost	2643 (43%) (1594 genes with altered intrinsic expression level^a^)	SQEC

^a^ Intrinsic expression level is expression level at t = 0, immediately before addition of toluene. The expression level was defined as ‘different’ at a ratio of <-0.5 or >0.5.

^b^ Toluene response is response immediately after addition of toluene until t = 30 min. The expression level was defined as ‘different’ when the correlation (of the expression pattern) between S12 and S12∆*trgI* was <0.8.

^c^ COGs were considered overrepresented if p<0.01 For COG abbreviations, see Fig 2.

## Results

### The dynamic response of *P*. *putida* S12 to sudden toluene exposure—global analysis of transcriptomics data

The addition of toluene to exponentially growing *P*. *putida* S12 provoked an extensive change in the global transcriptional activity (Fig 1). Although this effect was observed in both the wild-type and the *trgI*-knockout strain, marked differences were observed in the timing and the total number of differentially expressed genes. In wild-type *P*. *putida* S12, the abundance of differentially expressed genes increased immediately after toluene addition, reaching a maximum after 5 minutes, after which it decreased and more or less stabilized 10 min. after toluene addition. In *P*. *putida* S12∆*trgI*, the expression response appeared to be delayed. The abundance of differentially expressed genes was initially much lower and only reached a maximum 10 minutes after toluene addition. Subsequently, it stabilized to a level above that in the wildtype strain.

Furthermore, in Fig 1 the abundance of the genes is presented that were differentially expressed in both the wild-type strain and *P*. *putida* S12Δ*trgI*. This group likely represents genes responding to toluene irrespective of the presence of *trgI*. The abundance profile is remarkably similar to that of the differentially expressed genes in the *trgI*-knockout strain (Fig 1). The peak in the abundance profile of the wild-type strain 5 minutes after toluene addition is indicative of a generic rearrangement response that (partly) involves TrgI.

**Fig 2:**
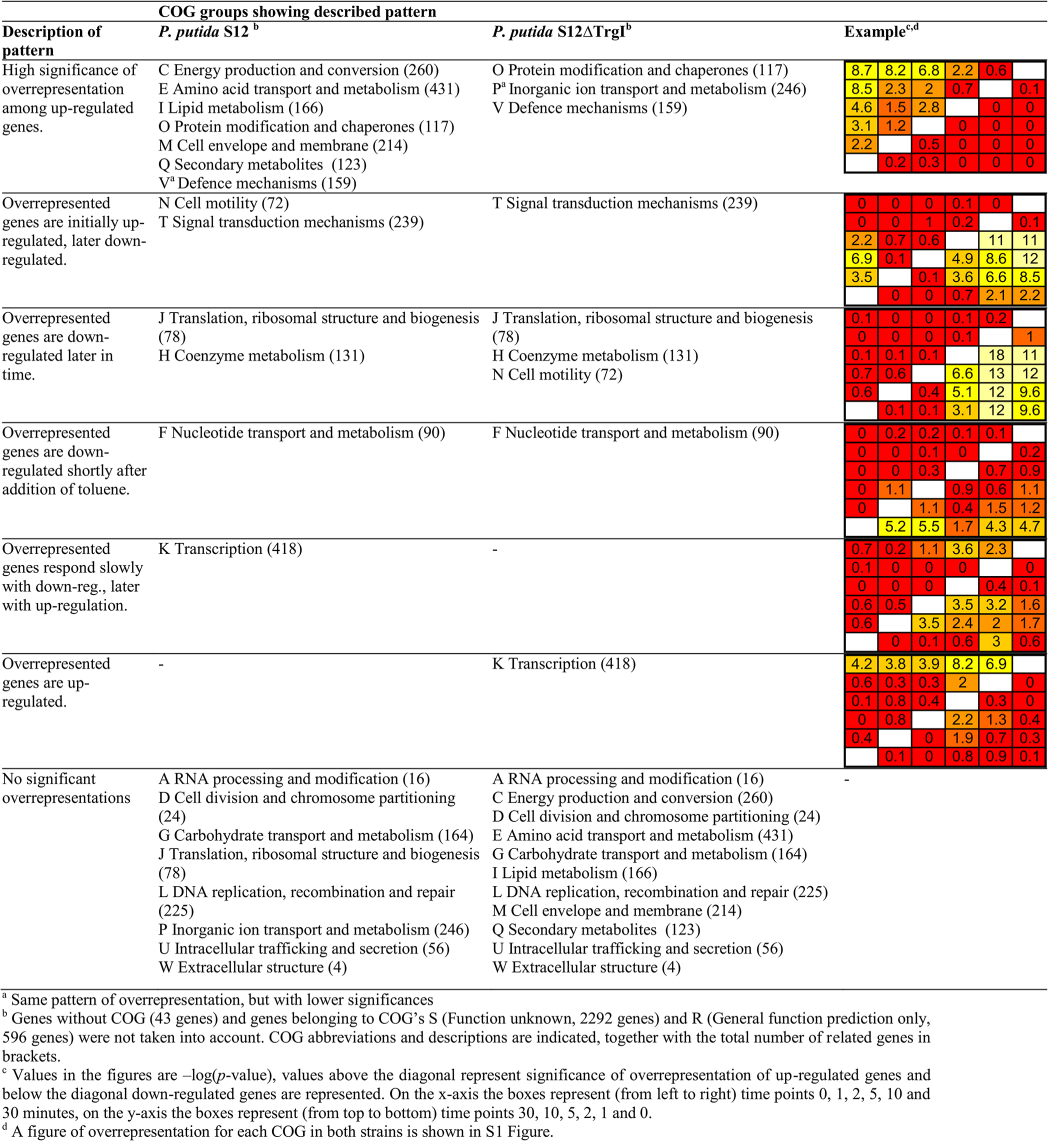
Overrepresentation of COG groups per time-period in *P*. *putida* S12 and *P*. *putida* S12∆*trgI*.

The fold changes of all genes whose expression altered after toluene addition were calculated for every possible combination of two time-points, *i*.*e*., between 0 and 1 minutes, 0 and 2 minutes, 5 and 30 minutes, etc. These combinations represent all possible time-frames within the experiment. The—apparently toluene-responsive—genes were furthermore classified by functional category to determine whether specific cellular functions were overrepresented within certain time frames. Functional classification was performed by the categories defined in the NCBI COG database (Clusters of Orthologous Groups of proteins; ftp://ftp.ncbi.nih.gov/pub/COG/COG/ [15,16]). Fig 2 shows a summary of the results for both wild-type *P*. *putida* S12 and the *trgI*-knockout strain; a complete overview is shown in S1 Fig.

Overrepresentation of specific functional categories was most explicit in wild-type *P*. *putida* S12, for genes up-regulated immediately after addition of toluene. Among these genes, category C (Energy production and conversion), E (Amino acid transport and metabolism), I (Lipid metabolism) and O (Posttranslational modification, protein turnover and chaperones) were significantly overrepresented. In *P*. *putida* S12∆*trgI* only category O was overrepresented among the genes that responded immediately to toluene exposure, consistent with the observed delayed global transcriptional response of S12Δ*trgI*.

Category T (signal transduction) was overrepresented among the genes that were first up-regulated and then down-regulated in response to toluene, in both wild-type *P*. *putida* S12 and S12Δ*trgI*. In the wild-type strain, also category N (motility) was overrepresented among these genes. In S12Δ*trgI*, category N was overrepresented among the genes that were down-regulated later after toluene addition.

Other categories overrepresented in both strains among this group of genes that were down-regulated later were J (translation) and H (coenzyme metabolism). Genes of category F (nucleotide transport and metabolism) were overrepresented in both strains early after toluene addition. In the wild-type strain, category K (transcription) genes were down-regulated a few minutes after addition of toluene, followed by up-regulation. In *P*. *putida* S12Δ*trgI*, genes of this category were among the genes that were consistently up-regulated after toluene addition. Thus, clear differences were visible between wild-type S12 and S12Δ*trgI*, both in the global transcriptional response to toluene and in the timing of expression of genes from different functional categories.

### Effect of *trgI* deletion on the transcriptional response to toluene

The analysis of the global transcriptional response to toluene revealed clear differences between wild-type *P*. *putida* S12 and strain S12Δ*trgI*. To establish which individual genes were affected by the *trgI* deletion, each gene was classified by expression behaviour (Table 1). The following criteria were applied: 1) expression response to toluene in wild-type *P*. *putida* S12; 2) expression level prior to toluene exposure; 3) expression response to toluene in *P*. *putida* S12∆*trgI*. Based on these criteria, four classes of genes can be discriminated by the effect of the *trgI* deletion on the intrinsic expression level and/or the response to toluene. Furthermore, a functional category (see above) was coupled to each individual gene to assess whether specific cellular functions were more affected than others by the *trgI* deletion (Table 1).

Only 1287 genes, *i*.*e*., around 20% of the total genome, were apparently unaffected by the *trgI* deletion with regard to both the intrinsic expression level and the response to toluene (group 1 in Table 1). Among these genes, particularly COGs O (posttranslational modification, protein turnover and chaperones), H (coenzyme metabolism) and D (cell division and chromosome partitioning) were overrepresented. This observation suggests that the *trgI*-deletion did not affect basic cell processes to a large extent. Also the number of genes that showed an unaltered response to toluene but an altered intrinsic expression level was relatively small (<500, group 2 in Table 1). Among these genes, COGs C (energy production and conversion) and O were overrepresented, indicating that the *trgI* deletion caused an intrinsic change in the energy metabolism, as well as in the chaperone machinery.

The large majority of the genes that were affected by the *trgI*-deletion showed an aberrant toluene response. These genes comprise a total of more than 4300 genes. More than 1700 genes appeared to have gained toluene responsiveness due to the *trgI* deletion (group 3 in Table 1). However, an even larger number (> 2600) appeared to have lost this property (group 4 in Table 1). The majority of this group (1594 genes) in addition showed an altered intrinsic expression level which may imply that these genes are expressed or repressed in a constitutive manner in *P*. *putida* S12∆*trgI*. Among the overrepresented functional groups in group 4, COG C can be linked to solvent tolerance which underlines the contribution of energy management systems to solvent tolerance.

### Effect of toluene and deletion of *trgI* on genes and pathways associated with toluene tolerance

As indicated above, deletion of *trgI* affected the expression of a very large number of genes, suggesting that *trgI* exerted a high-level regulatory effect. This regulatory effect was furthermore strongly associated with the response to toluene. Although the *trgI*-affected genes covered many different functional groups, COGs C, E and I (energy, amino acid metabolism and lipid metabolism) appeared to be overrepresented (Table 1 and Fig 2). Since these functional categories can be rationally linked to solvent tolerance, the transcriptional response of genes and pathways within these COGs was studied in more detail, together with genes and pathways with an established role in solvent tolerance (also see S1 Table).

### Toluene-responsive genes involved in energy metabolism

**Fig 3:**
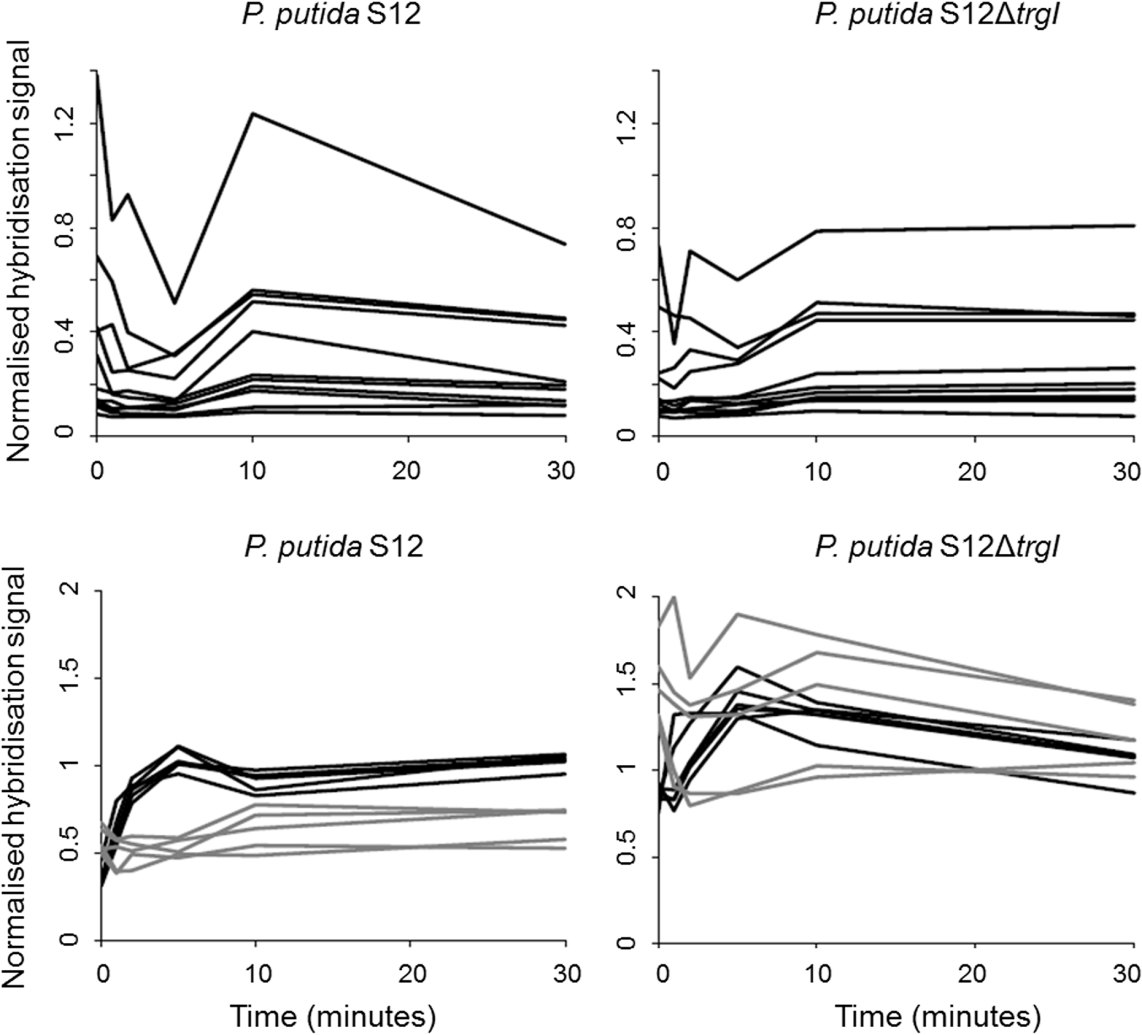
Normalised hybridisation signals of cytochrome *c* oxydase genes. Upper panels: cytochrome *c* oxidase aa_3_-type genes, Lower panels: cytochrome *c* oxidase cbb_3_-type genes. (black solid) cbb_3_-1, (grey) cbb_3_-2. (WT) *P*. *putida* S12; (KO) *P*. *putida* S12Δ*trgI*.

The accumulation of organic solvents in the bacterial membrane puts a heavy burden on the cellular energy metabolism as a result of uncoupling and the energy requirement of the solvent extrusion pump. This was clearly reflected in the differential expression of many genes associated with cellular energy management systems, most notably cytochrome *c* oxidase-encoding genes (Fig 3). *P*. *putida* S12 harbours several different types of terminal cytochrome *c* oxidases: in addition to the high-oxygen affinity types *cbb*_*3*_-1 and *cbb*_*3*_-2 there is also the low affinity variant *aa*_*3*_ [17].

The genes encoding the *aa*_*3*_-type were consistently expressed to a lower level than the genes encoding the *cbb*_*3*_-type, irrespective of the presence of toluene or *trgI*. The two *cbb*_*3*_ variants were expressed to different levels depending on the presence of toluene. In S12Δ*trgI*, the intrinsic expression level of both *cbb*_*3*_ type-encoding genes was higher than in the wild-type strain. In the presence of toluene, the expression level of the *cbb*_*3*_-1 genes increased even further and subsequently decreased to the wild-type level. The slightly variable expression levels of the *cbb*_*3*_-2 genes consistently exceeded those of wild-type *P*. *putida* S12 upon toluene exposure.

**Fig 4:**
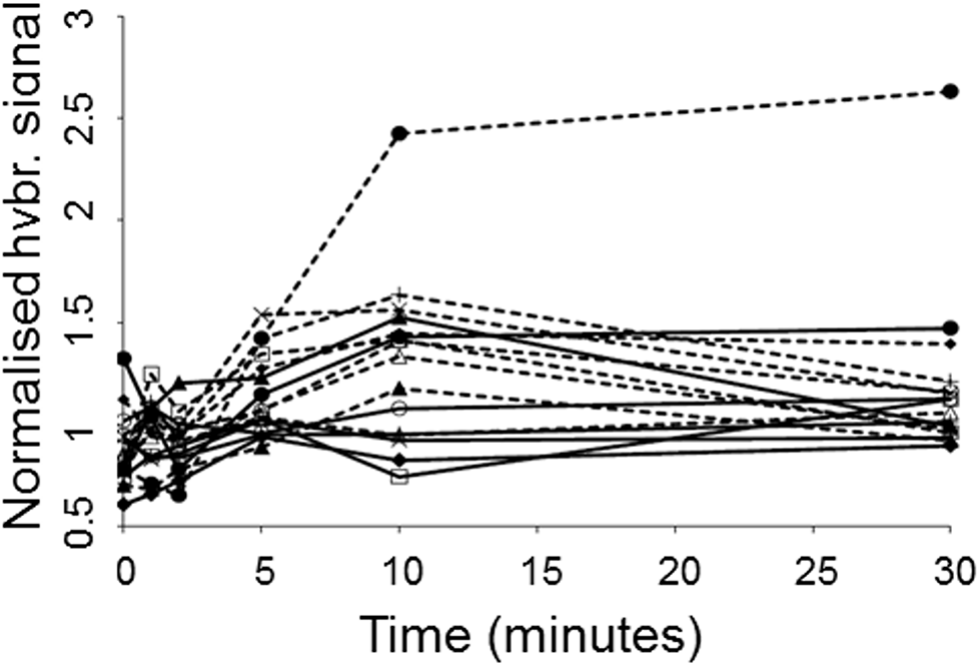
Normalised hybridisation signals of NADH dehydrogenase genes. Solid lines: *P*. *putida* S12; dotted lines: *P*. *putida* S12Δ*trgI*. ● NADH dehydrogenase (probe PS1205605_at), ♦ NADH dehydrogenase (probe PS1200059_at), ▲ NADH-quinine oxidoreductases chain A, × NADH-quinone oxidoreductases chain B, + NADH-quinone oxidoreductases chain C, ○ NADH-quinone oxidoreductases chain E, ∆ NADH-quinone oxidoreductases chain I, □ NADH-quinone oxidoreductases chain L.

Along with cytochrome *c* oxidase-encoding genes, several genes encoding subunits of the NADH dehydrogenase complex were expressed to a higher level in S12Δ*trgI* within 10 minutes after toluene exposure (Fig 4). After 30 minutes, the expression level of the genes encoding the quinone oxidoreductase-subunit had returned to the wild-type level whereas the genes coding for the dehydrogenase subunit remained at a high level for at least 30 minutes after toluene exposure.

### Toluene-responsive genes involved in lipid metabolism

The accumulation of organic solvent molecules in the bacterial membrane leads to cell lysis, releasing membrane fatty acids into the medium. In *E*. *coli* free fatty acids induce fatty acid degradation via *fadR* [18]. *P*. *putida* S12 does not harbour a *fadR* homologue, but the gene *psrA* is responsible for the regulation of part of the fatty acid degradation pathway [18,19]. In toluene-exposed *P*. *putida* S12 *psrA* as well as the genes of the fatty acid degradation pathway were up-regulated after addition of toluene (S2 Fig, solid lines). In addition to fatty acid degradation, fatty acid synthesis is expected to occur in solvent-exposed *P*. *putida* S12 since newly synthesized fatty acids are required to counteract the toluene-induced degradation effects [20]. Differential expression of genes involved in fatty acid biosynthesis, however, was variable (S3 Fig). In wild-type *P*. *putida* S12, COG I genes were overrepresented after addition of toluene. In S12Δ*trgI*, this was not the case (Fig 2) indicating less toluene-induced membrane damage in this strain, or a lower need for *de-novo* membrane fatty acid synthesis under toluene stress. It should be noted that the fatty acid biosynthetic genes are poorly annotated in the *P*. *putida* S12 genome that was used for the custom micro array, which prevented a more detailed analysis of the responses of these genes to toluene.

### Toluene-responsive genes involved in acetyl-CoA metabolism

The genes encoding isocitrate lyase and malate synthase were found to be up-regulated in response to toluene. This effect was observed in both wild-type S12 and S12Δ*trgI* (S4 Fig) although the level of up-regulation was higher in the latter. The encoded enzymes constitute the glyoxylate bypass, which converts acetyl-CoA into succinate and malate for biosynthesis of, a.o., amino acids of the aspartate family. This pathway is normally active during growth on C_2_-compounds and is controlled at the enzymatic level through NADH-mediated inhibition of isocitrate dehydrogenase [21]. Generally, induction of the glyoxylate bypass genes and bypass activity are correlated [22], suggesting that NADH is present at surplus amounts during toluene stress. This may be unexpected in view of the high energy demand brought about by toluene stress, but it may also be an effect caused by malfunctioning respiratory chain components. It is well known that the functioning of membrane-embedded proteins is affected by accumulation of solvent molecules in the cytoplasmic membrane, but also due to compositional changes of the membrane in response to solvents [23]. Possibly, the differential expression of respiratory chain components in response to toluene (as described above) is a compensatory response to compromised electron transport chain functioning. Alternatively, the glyoxylate pathway may be co-regulated with the degradation of fatty acids, or with valine, leucine and isoleucine degradation. In these pathways, the main degradation product is acetyl-CoA which effectively represents a C_2_ substrate [18]. It should be noted that multiple genes involved in the degradation of these amino acids were up-regulated in response to toluene and, again, the level of up-regulation was significantly higher in S12Δ*trgI* (S5 Fig).

### Toluene-responsive genes involved in solvent extrusion

**Fig 5:**
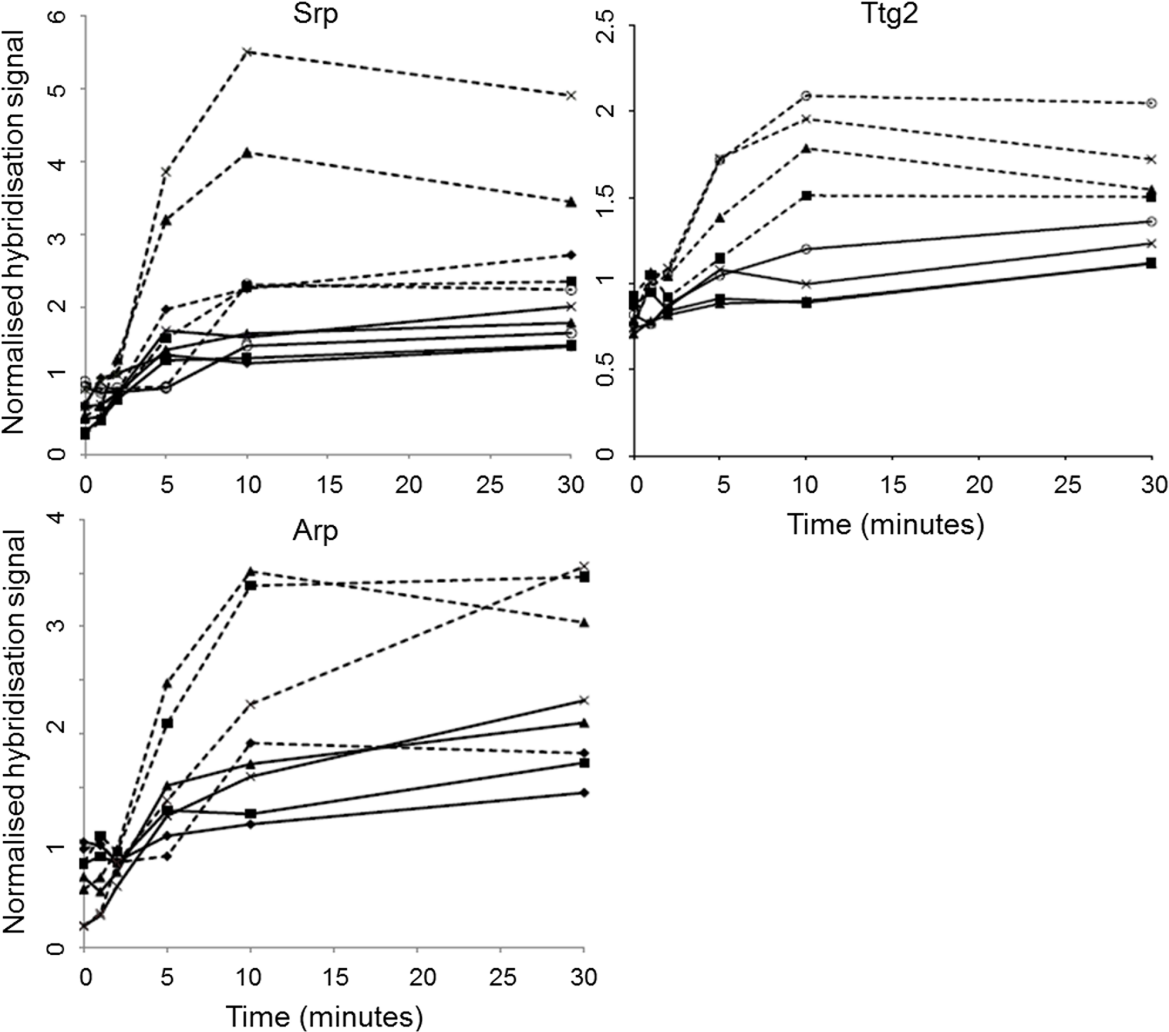
Normalised hybridisation signals of *srpRSABC* and *ttg2DC* and the genes coding for a toluene transport system permease and ATP-binding protein. Solid lines: *P*. *putida* S12; dotted lines: *P*. *putida* S12Δ*trgI*. Upper left panel: ♦ *srpR*; ■ *srpS*; ▲ *srpA*; × *srpB*; ○ *srpC*. Upper right panel: ▲ *ttg2C*; ■ *ttg2D*; × toluene transport system permease; ○ toluene transport system ATP-binding protein. Lower left panel: ▲ *arpA*; ■ *arpB*; ♦ *arpC*; × *arpR*.

Expression of *srpABC*, coding for the solvent extrusion pump SrpABC, and *srpRS*, the associated regulatory genes, increased immediately in wild type *P*. *putida* S12 after addition of toluene. This is in accordance with previous observations [7], although the expression level appeared to stabilize after 5 min. of toluene exposure rather than reaching a maximum after 30 min. as reported earlier [24] (Fig 5). Expression of other loci, encoding part of the putative toluene transport system Ttg2FEDC and the multidrug transporter ArpABC, increased immediately after addition of toluene, reaching maximum levels after 10 to 30 minutes of toluene exposure (Fig 5). All expression levels were similar in wild-type and S12Δ*trgI* prior to toluene exposure, but increased much faster in the mutant, to much higher levels. It is unclear to which extent Ttg2FEDC is involved in toluene tolerance, and ArpABC has been established to play a role in antibiotic resistance rather than solvent tolerance [25]. These observations indicate that *trgI* is involved in modulation of general responses to stress conditions including, but not restricted to, solvent-associated stress. These observations clearly indicate that, next to solvent-associated stress, *trgI* is involved in modulation of other stress responses as well.

### Effect of toluene and deletion of *trgI* on genes and pathways that are not directly associated with toluene tolerance

#### Differential expression of the arginine deiminase pathway in response to toluene

The ubiquitous arginine deiminase (ADI) pathway serves to generate energy from arginine fermentation under anoxic or carbon-limiting conditions [26]. In the ADI pathway, arginine degradation via citrulline to ornithine yields 1 ATP per arginine molecule. Each ornithine generated is exported and exchanged for another arginine molecule via an arginine-ornithine specific antiporter. In wild-type *P*. *putida* S12, the genes encoding the enzymes for this pathway (arginine deiminase, ornithine carbamoyltransferase and carbamate kinase) and the arginine/ornithine antiporter were up-regulated immediately after addition of toluene (S6 Fig). In contrast, another associated arginine/ornithine antiporter gene appeared to be down-regulated, together with *aotM*, *aotP* and *aotQ*, that constitute an arginine/ornithine importer. In *P*. *putida* S12Δ*trgI* the expression of the ADI pathway genes was considerably higher; already in the absence of toluene, the expression was similar to that of the wild type after 30 minutes of toluene exposure. Upon addition of toluene, the expression levels increased even further. The expression of the arginine/ornithine antiporter gene decreased in strain S12Δ*trgI* after an initial up-regulation, as did the expression of *aotM*, *aotP* and *aotQ*. Since the ADI pathway genes were up-regulated while the antiporter genes were down-regulated, it appears that the ADI pathway rather serves to accumulate intracellular ornithine than to generate energy under toluene-stressed conditions.

#### Differential expression of protein folding genes in response to toluene

In the periplasm, proteins can be folded oxidatively via disulphide bond formation. The enzymes involved in this process are DsbAB, which oxidizes unfolded proteins and DsbCDG, which isomerizes disulfide bonds in misfolded proteins [27]. In *P*. *putida* S12, *dsbACDG* have been annotated, as well as *tlpA* that likely also belongs to this system. In addition, chaperonins like DnaJ, DnaK, GroES, GroEL and HtpG play an important role in protein folding. All genes involved in this protein-damage repair system as well as a gene encoding a 33-kDa chaperonin were down-regulated in the wild-type strain within the first two minutes of toluene exposure, and subsequently up-regulated (S7 Fig and S8 Fig). In S12Δ*trgI*, most if not all genes were up-regulated to an even higher extent. Hence, the protein damage-repair response is induced even more strongly in strain S12∆*trgI* during solvent exposure.

### Deletion of *trgI* affects glucose and fructose metabolism

A remarkable physiological effect of the *trgI* deletion is loss of the ability to utilize glucose or fructose as the sole source of carbon and energy [6]. Whereas fructose is imported via a PTS-type transporter and further metabolized in the cytoplasm as fructose-1-phosphate, the initial glucose metabolism is more complex in *Pseudomonads*. Glucose enters the periplasm via porins OprB-1 or OprB-2 and is subsequently transported directly into the cytoplasm via an ABC transporter encoded by *gtsABCD*, or oxidized in the periplasm via gluconate (catalyzed by Gcd, glucose dehydrogenase) to 2-ketogluconate (catalysed by Gad) [28]. Gluconate and 2-ketogluconate are transported to the cytoplasm via dedicated transporters encoded by *gntP*, respectively *kguT* and phosphorylated by GnuK, respectively KguK.

**Fig 6:**
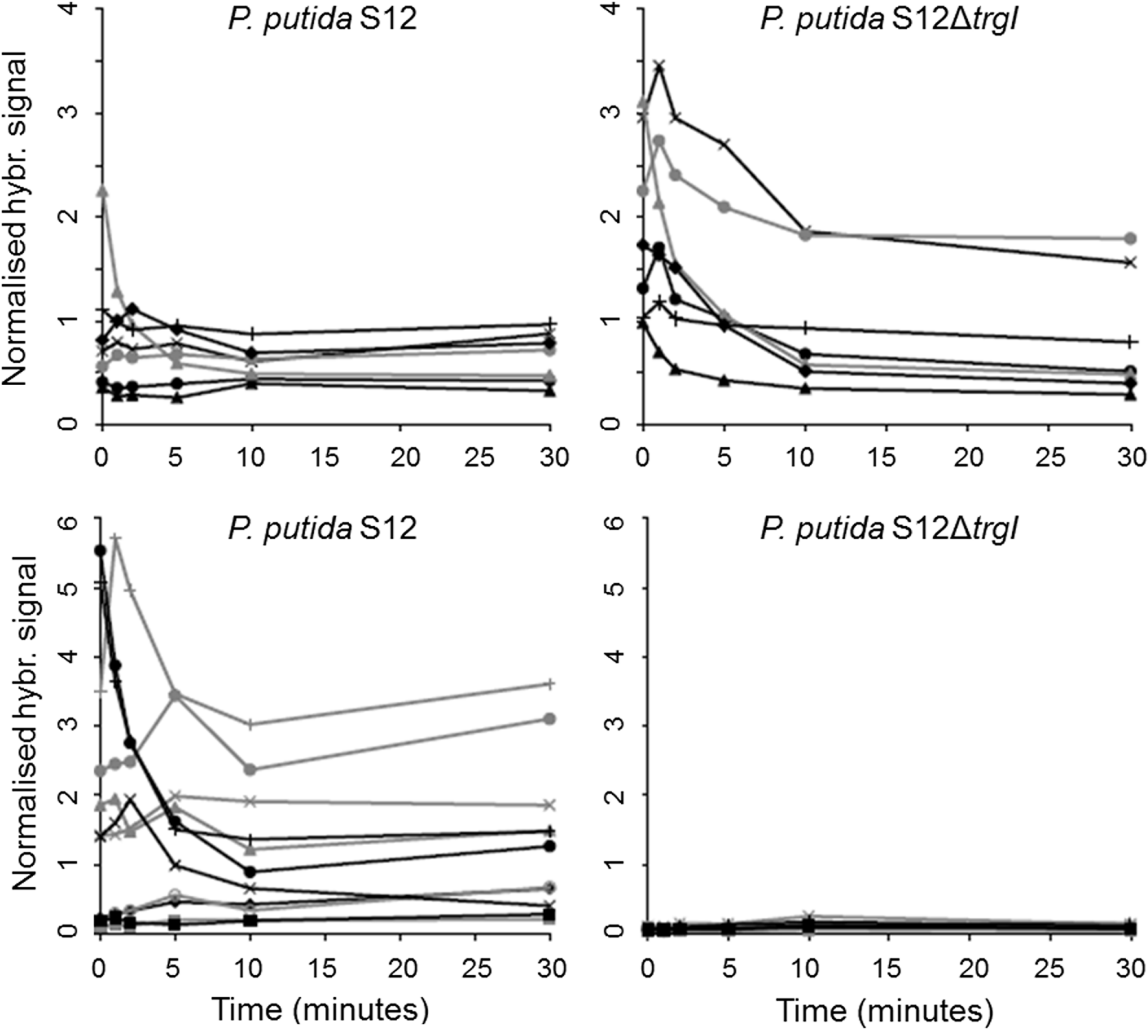
Normalised hybridisation signals of genes involved in glucose import and metabolism. (Upper panels) ♦ *gtsA*, ▲*gtsB*, ● *gtsC*, × *gtsD*, + *gcd*, ▲ *oprB-2*, ● *oprB-1*; (Lower panels) + *ptxS*, ○ *kguK*, ■ *kguT*, × *gad* cytochrome c subunit, ● *gad* alpha chain, ▲ *gad* gamma chain, + *gnuK*, ● *gntP*, × *gnuR*, ♦ *kguD*, ■ σ54-dependent transcriptional regulator.

The expression of the fructose transporter genes was similar in wild-type *P*. *putida* S12 and in strain S12∆*trgI* whereas transcript levels of *oprB-1*, *oprB-2* and *gtsABCD* were actually higher in S12Δ*trgI* (Fig 6). *Gcd* was expressed at a slightly lower level whereas *gad*, *gntP*, *gnuK*, *kguT* and *kguK* were not detectably transcribed. The expression of these genes is known to be induced by gluconate and ketogluconate [29,30]. Therefore, the lack of detectable expression suggested that gluconate and ketogluconate could not be formed. Since both *gcd* and *gtsABCD* (as well as the fructose transporter genes) were transcribed, impaired transport of glucose (and fructose) into the periplasm appears to be the only viable explanation for the lack of growth on sugars. This phenomenon may be caused by structural modification of the outer cell structure, which may affect the functioning of the membrane-embedded transport proteins. A similar explanation has been reported for the impaired functioning of drugs efflux transporters of a mutant *P*. *putida* DOT-T1E strain with altered phospholipid head group composition [31].

## Discussion

*TrgI* is a previously identified toluene-responsive gene of *P*. *putida* S12 with unknown function [7]. To gain further insight into the solvent tolerance response mechanism of *Pseudomonas* and into a possible role of TrgI, the transcriptome responses of wild type S12 and of *trgI*-deletion mutant S12Δ*trgI* [7] were monitored after addition of toluene.

In the first five minutes after toluene addition, the absolute number of differentially expressed genes was significantly smaller in a ∆*trgI* strain than in a wild-type strain. Many genes in the mutant showed an altered intrinsic expression level. This suggested that TrgI is not only involved in toluene-elicited responses but also affects basal expression levels of large groups of genes. It appears that inactivation of *trgI* prepares the cells for a sudden addition of organic solvent by ‘setting’ basal expression of many genes to levels comparable to those in toluene-exposed wild type cells. However, the number of differentially expressed genes remained high in strain S12Δ*trgI*. Thus, some feedback loop appears to be disturbed as well by the deletion of *trgI*.

NADH dehydrogenase- and cytochrome *c* oxidase genes were differentially expressed upon toluene exposure, as well as more structurally in the *trgI*-deletion mutant. This fully correlated with the proton gradient-dissipating effect of toluene [33,34]. The relatively high expression of the cbb_3_-type cytochrome oxidase over the lower oxygen-affinity aa_3_-type in both wild-type and mutant strains suggested that in cultures exposed to toluene, high-affinity cytochrome *c* oxidase activity is specifically needed to provide for an adequate proton gradient.

Cells exposed to organic solvents are known to change their fatty acid composition to strengthen their membranes by increasing the degree of saturation by cis-trans isomerisation of unsaturated fatty acids or by newly synthesising long chain fatty acids [20,32,35]. Eventually, accumulation of solvent molecules in the membranes results in cell lysis. Lysing cells release fatty acids that trigger expression of the genes encoding the fatty acid degradation pathway [33]. However, we did not find evidence that fatty acid-synthesis was increased. This suggested that the capacity of the available fatty acid synthesis machinery was sufficient.

Genes encoding the Ttg2FEDC putative toluene transport system were rapidly up-regulated in response to toluene in *P*. *putida* S12. Previously, only the SrpABC solvent efflux pump was found to be specifically expressed in response to toluene [2,7]. As SrpABC becomes fully expressed only after extended toluene exposure [25], Ttg2FEDC may cushion the first blow dealt by the sudden exposure to toluene. In *P*. *putida* S12Δ*trgI* maximum expression levels of the Srp genes was reached much sooner, as with the Ttg2 genes and the genes coding for the antibiotic pump ArpABC.

Remarkably, the ADI (arginine deiminase) pathway appeared to be up-regulated in response to toluene, and to an even higher level in S12Δ*trgI* than in the wildtype strain. Only one arginine-ornithine antiporter gene was down-regulated in the wild-type whereas both were down-regulated in S12Δ*trgI*, suggesting that *P*. *putida* S12Δ*trgI* accumulates the polyamine ornithine in response to toluene, providing additional protection against solvent stress [34]. Moreover, ornithine may act as a precursor for proline, a compatible solute implicated in protection against water and solvent stress [36,37,38]. Since the cultures were in mid-exponential phase, it is unlikely that either anoxic or carbon-limiting conditions occurred, which are the normal conditions for inducing the ADI pathway [26].

Genes involved in oxidative protein folding and several chaperonin-encoding genes were up-regulated in both wild-type *P*. *putida* S12 and S12Δ*trgI* upon addition of toluene. Presumably, the up-regulation of those genes related to the occurrence of protein misfolding and random formation of disulfide bonds. These events may well occur in solvent-exposed cells as a result of oxidative stress caused by impaired respiration. Protein folding genes were up-regulated to a higher level in the *trgI*-knockout strain, suggesting this strain has a higher need of them.

Transcriptome analysis of *P*. *putida* S12 and S12∆*trgI* following sudden exposure to toluene has shed new light on solvent tolerance. The initial response to toluene in wild-type cells appeared to be fully geared towards survival before all solvent tolerance mechanisms are fully induced. The clear delay in timing of the overall transcriptional response in the *trgI*-deletion mutant was indicative for a milder response to sudden toluene exposure. This would be in agreement with an intrinsically improved toluene tolerance. The impact of the *trgI* deletion thus appeared to be relatively broad.

The diversity of responses associated with *trgI*, as well as its wide impact on gene expression, suggest an important regulatory role. Analyses using BLAST (http://blast.ncbi.nlm.nih.gov/Blast.cgi) and SMART (http://smart.embl-heidelberg.de/) did not provide any clues to a potential function. A model of TrgI tertiary structure (http://zhanglab.ccmb.med.umich.edu/I-TASSER/) [39] showed highest structural similarity (TM-score of 0.646) with a molybdate-dependent transcriptional regulator, *modE*, of *E*. *coli* [40], although TrgI is much smaller than ModE and does not exhibit clear DNA binding residues. Hence, one hypothesis may envision TrgI as a modulator of other transcription factors.

## Supporting Information

S1 Fig Overrepresentation of COG groups per time-period in *P*. *putida* S12 (upper panel) and *P*. *putida* S12∆*trgI* (lower panel). Values are –log(*p*-value). Values above the diagonal represent significance of overrepresentation of up-regulated genes and below the diagonal down-regulated genes are represented. Genes without COG (43 genes) and genes belonging to COG’s S (Function unknown, 2292 genes) and R (General function prediction only, 596 genes) are not shown. Abbreviations (with total amount of genes in brackets): A RNA processing and modification (16), C Energy production and conversion (260), D Cell division and chromosome partitioning (24), E Amino acid transport and metabolism (431), F Nucleotide transport and metabolism (90), G Carbohydrate transport and metabolism (164), H Coenzyme metabolism (131), I Lipid metabolism (166), J Translation, ribosomal structure and biogenesis (78), K Transcription (418), L DNA replication, recombination and repair (225), M Cell envelope biogenesis and outer membrane (214), N Cell motility (72), O Posttranslational modification, protein turnover and chaperones (117), P Inorganic ion transport and metabolism (246), Q Secondary metabolites biosynthesis, transport and catabolism (123), T Signal transduction mechanisms (239), U Intracellular trafficking and secretion (56), V Defence mechanisms (159), W Extracellular structure (4).
(PDF)

S2 Fig Normalised hybridisation signals of genes of the fatty acid degradation pathway. Solid lines: *P*. *putida* S12; Dotted lines *P*. *putida* S12Δ*trgI*. (A) 3-ketoacyl-CoA thiolase; (B) ■ acetyl-CoA acetyltransferase and ▲ acyl-CoA dehydrogenase (1.3.99.-); (C) acyl-CoA dehydrogenase (EC 1.3.99.3); (D) ♦ acyl-CoA dehydrogenase, short-chain specific and ● alcohol dehydrogenase; (E) aldehyde dehydrogenase; (F) Δ enoyl-CoA hydratase/delta(3)-cis-delta(2)-trans-enoyl-CoA isomerase/3-hydroxyacyl-CoA dehydrogenase/3-hydroxybutyryl-CoA epimerase, ○ enoyl-CoA hydratase, ◊ glutaryl-CoA dehydrogenase and □ glutarate-CoA ligase; (G) long chain fatty acid-CoA ligase; (H) × membrane-bound aldehyde dehydrogenase iron-sulfur protein and + rubredoxin-NAD(+) reductase.
(PDF)

S3 Fig Normalised hybridisation signals of genes involved in the biosynthesis of fatty acids. Upper panels: ♦ *fabH* (probe PS1201104_at), ■ *fabH* (probe PS1201105_at), ▲ *fabH* (probe PS1209460_at), ◊ *fabD*, ● biotin carboxylase (probe PS1206980_at), - biotin carboxylase (probe PS1202405_at), ∆ Biotin carboxyl carrier protein of acetyl-CoA carboxylase, × Acetyl-coenzyme A carboxylase carboxyl transferase subunit alpha, □ Acetyl-coenzyme A carboxylase carboxyl transferase subunit beta; Lower panels: ■ *fabG* (probe PS1201047_at), ● *fabG* (probe PS1204926_at), ▲ *fabG* (probe PS1201366_at), ♦ *fabG* (probe PS1200008_at), □ *fabG* (probe PS1204304_at), × *fabG* (probe PS1203087_at), + *fabB* (probe PS1200004_at), ◊ *fabB* (probe PS1201484_at).
(PDF)

S4 Fig Normalised hybridisation signals of the genes of the glyoxylate shunt. Solid lines: *P*. *putida* S12, dotted lines: *P*. *putida* S12Δ*trgI*. ■ malate synthase, ♦ isocitrate lyase.
(PDF)

S5 Fig Normalised hybridisation signals of genes of the valine, leucine and isoleucine degradation pathway. Solid lines: *P*. *putida* S12; Dotted lines *P*. *putida* S12Δ*trgI*. (A) ■ 3-hydroxyisobutyrate dehydrogenase, ▲ leucine dehydrogenase, ♦ 3-hydroxyacyl CoA dehydrogenase, ● 2-oxoisovalerate dehydrogenase alpha or beta subunit; (B) aldehyde dehydrogenase; (C) Δ isovaleryl-CoA dehydrogenase, ◊ acyl-CoA dehydrogenase, short-chain specific, □ acyl-CoA dehydrogenase (EC 1.3.99.-); (D) acyl-CoA dehydrogenase (EC 1.3.99.3); (E) 3-ketoacyl-CoA thiolase; (F) × omega-amino acid—pyruvate aminotransferase, + dihydrolipoamide dehydrogenase, ○ acetyl-CoA acetyltransferase; (G) enoyl-CoA hydratase, ● enoyl-CoA hydratase / delta(3)-cis-delta(2)-trans-enoyl-CoA isomerase / 3-hydroxyacyl-CoA dehydrogenase / 3-hydroxybutyryl-CoA epimerase; (H) (Grey symbols) ● branched-chain amino acid aminotransferase, Δ methylcrotonyl-CoA carboxylase carboxyl transferase subunit, □ 3-hydroxyisobutyryl-CoA hydrolase, ◊ hydroxymethylglutaryl-CoA lyase, ♦ succinyl-CoA:3-ketoacid-coenzyme A transferase subunit A, ▲ methylcrotonyl-CoA carboxylase biotin-containing subunit, ■ methylglutaconyl-CoA hydratase.
(PDF)

S6 FigNormalised hybridisation signal of genes coding for the ADI pathway and arginine/ornithine transporter genes.Black symbols: ♦ arginine deiminase, ▲ornithine carbamoyltransferase, ■ ornithine carbamoyltransferase, ● carbamate kinase, × carbamate kinase, ∆ ornithine cyclodeaminase, □ ornithine cyclodeaminase family protein; Grey symbols: ▲ *aotM*, × *aotP*, ● *aotQ*, ♦ arginine/ornithine antiporter, ■ arginine/ornithine antiporter.(PDF)

S7 FigNormalised hybridisation signals of the oxidative protein folding genes.Black symbols: ♦ *dsbA*, ▲*dsbD*, ■ *dsbC*, ○ RPPX01161 *tlpA*, ● RPPX04273 *tlpA*; grey symbols: ♦ RPPX05428 *dsbG*, ▲ RPPX04274 *dsbG*, ■ *dsbD*.(PDF)

S8 Fig Normalised hybridisation signals of chaperonin genes. Solid lines: *P*. *putida* S12; Dotted lines *P*. *putida* S12Δ*trgI*. ♦ *groEL*, ▲ *groES*, ● 33 kDa chaperonin (RPPX07096), ■ *dnaJ*, □ *htpG* (RPPX05623), ○ *htpG* (RPPX05624).
(PDF)

S1 Table Overview of all genes mentioned in main text. To make it easier to find all genes mentioned in the text in the GenBank and GEO databases, these genes are shown with their names, RPPX numbers, probe names, and additional remarks.
(XLSX)

## References

[1] Inoue A, Horikoshi K. A *Pseudomonas* thrives in high concentrations of toluene. Nature. 1989;338: 264–266.

[2] Volkers RJ, Ballerstedt H, de Winde JH, Ruijssenaars H. Isolation and genetic characterization of an improved benzene tolerant mutant of *Pseudomonas putida* S12 Environ Microbiol Reports. 2010;2(3): 456–460. 10.1111/j.1758-2229.2010.00172.x 23766120

[3] Isken S, de Bont JAM. Bacteria tolerant to organic solvents. Extremophiles. 1998;2: 229–238. 9783170 10.1007/s007920050065

[4] Kuepper J, Ruijssenaars HJ, Blank LM, De Winde JH, Wierckx N. Complete genome sequence of solvent-tolerant *Pseudomonas putida* S12 including megaplasmid pTTS12. J Biotechnol. 2015;200: 17–18. 10.1016/j.jbiotec.2015.02.027 25746905

[5] Isken S, Derks A, Wolffs PFF, de Bont JAM. Effect of organic solvents on the yield of solvent-tolerant *Pseudomonas putida* S12. Appl Environ Microbiol. 1999;65: 2631–2635. 10347053 10.1128/aem.65.6.2631-2635.1999 PMC91388

[6] Kieboom J, Dennis JJ, de Bont JAM, Zylstra G. Identification and molecular characterization of an efflux pump involved in *Pseudomonas putida* S12 solvent tolerance. J Biol Chem. 1998;273: 85–91. 9417051 10.1074/jbc.273.1.85

[7] Volkers RJ, Ballerstedt H, Ruijssenaars H, de Bont JAM, de Winde JH, Wery J. TrgI, toluene repressed gene I, a novel gene involved in toluene-tolerance in *Pseudomonas putida* S12. Extremophiles. 2009;13: 283–297. 10.1007/s00792-008-0216-0 19089528

[8] Volkers RJM, de Jong AL, Hulst AG, van Baar BLM, de Bont JAM, Wery J. Chemostat-based proteomic analysis of toluene-affected *Pseudomonas putida* S12. Environ Microbiol. 2006;8: 1674–1679. 16913927 10.1111/j.1462-2920.2006.01056.x

[9] Trautwein K, Kuhner S, Wohlbrand L, Halder T, Kuchta K, Steinbüchel A et al Solvent stress response of the denitrifying bacterium "*Aromatoleum aromaticum*" strain EbN1. Appl Environ Microbiol. 2008;74: 2267–2274. 10.1128/AEM.02381-07 18263750 PMC2293168

[10] He MX, Wu B, Shui ZX, Hu QC, Wang WG, Tan FR, et al Transcriptome profiling of *Zymomonas mobilis* under ethanol stress. Biotechnol Biofuels. 2012;5: 75 10.1186/1754-6834-5-75 23057803 PMC3495753

[11] Lauchnor EG, Radniecki TS, Semprini L. Inhibition and gene expression of *Nitrosomonas europaea* biofilms exposed to phenol and toluene. Biotechnol Bioeng. 2011;108: 750–757. 10.1002/bit.22999 21404249

[12] Muller EE, Hourcade E, Louhichi-Jelail Y, Hammann P, Vuilleumier S, Bringel F. Functional genomics of dichloromethane utilization in *Methylobacterium extorquens* DM4. Environ Microbiol. 2011;13: 2518–2535. 10.1111/j.1462-2920.2011.02524.x 21854516

[13] Hartmans S, Smits J, van de Werf M, Volkering F, de Bont JAM. Metabolism of styrene oxide and 2-phenyl ethanol in the styrene degrading *Xanthobacter* strain 124X. Appl Environ Microbiol. 1989;55: 2850–2855. 16348047 10.1128/aem.55.11.2850-2855.1989 PMC203180

[14] Sambrook J, Fritsch EF, Maniatis T. Molecular Cloning: A Laboratory Manual. Cold Spring Harbor: CSHL Press; 1982.

[15] Tatusov RL, Fedorova ND, Jackson JD, Jacobs AR, Kiryutin B, Koonin EV, et al The COG database: an updated version includes eukaryotes. BMC Bioinformatics. 2003;4: 41 12969510 10.1186/1471-2105-4-41 PMC222959

[16] Tatusov RL, Koonin EV, Lipman DJ. A genomic perspective on protein families. Science. 1997;278: 631–637. 9381173 10.1126/science.278.5338.631

[17] Arai H, Kawakami T, Osamura T, Hirai T, Sakai Y, Ishii M. Enzymatic characterization and in-vivo function of five terminal oxidases in *Pseudomonas aeruginosa* , J Bacteriol. 2014;196: 4206–15. 25182500 10.1128/JB.02176-14 PMC4248849

[18] Kazakov AE, Rodionov DA, Alm E, Arkin AP, Dubchak I, Gelfand MS. Comparative genomics of regulation of fatty acid and branched-chain amino acid utilization in proteobacteria. J Bacteriol. 2009;191: 52–64. 10.1128/JB.01175-08 18820024 PMC2612455

[19] Fonseca P, de la Peña F, Prieto MA. A role for the regulator PsrA in the polyhydroxyalkanoate metabolism of *Pseudomonas putida* KT2440. Int J Biol Macromol. 2014;71: 14–20. 10.1016/j.ijbiomac.2014.04.014 24751507

[20] Heipieper HJ, Neumann G, Cornelissen S, Meinhardt F. Solvent-tolerant bacteria for biotransformations in two-phase fermentation systems. Appl Microbiol Biotechnol. 2007;74: 961–973. 17262209 10.1007/s00253-006-0833-4

[21] Berg JM, Tymoczko JL, Stryer L. Biochemistry. New York: W.H. Freeman and Company; 2002.

[22] Sunnarborg A, Klumpp D, Chung T, LaPorte DC. Regulation of the glyoxylate bypass operon: cloning and characterization of iclR. J Bacteriol. 1990;172: 2642–2649. 2185227 10.1128/jb.172.5.2642-2649.1990 PMC208908

[23] Ramos JL, Duque E, Gallegos MT, Godoy P, Ramos-Gonzalez MI, Rojas A, et al Mechanisms of solvent tolerance in gram-negative bacteria. Annu Rev Microbiol. 2002;56: 743–768. 12142492 10.1146/annurev.micro.56.012302.161038

[24] Kieboom J, Dennis JJ, Zylstra GJ, de Bont JAM. Active efflux of organic solvents by *Pseudomonas putida* S12 is induced by solvents. J Bacteriol. 1998;180: 6769–6772. 9852029 10.1128/jb.180.24.6769-6772.1998 PMC107788

[25] Kieboom J, de Bont JAM. Identification and molecular characterization of an efflux system involved in *Pseudomonas putida* S12 multidrug resistance. Microbiology. 2001;147: 43–51. 11160799 10.1099/00221287-147-1-43

[26] Mercenier A, Simon J-P, Vander Wauven C, Haas D, Stalon V. Regulation of enzyme synthesis in the arginine deiminase pathway of *Pseudomonas aeruginosa* . J Bacteriol. 1980;144: 159–163. 6252188 10.1128/jb.144.1.159-163.1980 PMC294610

[27] Collet JF, Bardwell JC. Oxidative protein folding in bacteria. Mol Microbiol. 2002;44: 1–8. 11967064 10.1046/j.1365-2958.2002.02851.x

[28] del Castillo T, Ramos JL, Rodriguez-Herva JJ, Fuhrer T, Sauer U, Duque E. Convergent peripheral pathways catalyze initial glucose catabolism in *Pseudomonas putida*: genomic and flux analysis. J Bacteriol. 2007;189: 5142–5152. 17483213 10.1128/JB.00203-07 PMC1951859

[29] Frunzke J, Engels V, Hasenbein S, Gatgens C, Bott M. Co-ordinated regulation of gluconate catabolism and glucose uptake in *Corynebacterium glutamicum* by two functionally equivalent transcriptional regulators, GntR1 and GntR2. Mol Microbiol. 2008;67: 305–322. 18047570 10.1111/j.1365-2958.2007.06020.x PMC2230225

[30] Swanson BL, Hager P, Phibbs P Jr., Ochsner U, Vasil ML, Hamood AN. Characterization of the 2-ketogluconate utilization operon in *Pseudomonas aeruginosa* PAO1. Mol Microbiol. 2000;37: 561–573. 10931350 10.1046/j.1365-2958.2000.02012.x

[31] Bernal P, Segura A, Ramos JL. Compensatory role of the cis-trans-isomerase and cardiolipin synthase in the membrane fluidity of *Pseudomonas putida* DOT-T1E. Environ Microbiol. 2007;9: 1658–1664. 17564601 10.1111/j.1462-2920.2007.01283.x

[32] Zhang YM, Rock CO. Membrane lipid homeostasis in bacteria. Nat Rev Microbiol. 2008;6: 222–233. 10.1038/nrmicro1839 18264115

[33] DiRusso CC, Heimert TL, Metzger AK. Characterization of FadR, a global transcriptional regulator of fatty acid metabolism in *Escherichia coli*. Interaction with the fadB promoter is prevented by long chain fatty acyl coenzyme A. J Biol Chem. 1992;267: 8685–8691. 1569108

[34] Rhee HJ, Kim EJ, Lee JK. Physiological polyamines: simple primordial stress molecules. J Cell Mol Med. 2007;11: 685–703. 17760833 10.1111/j.1582-4934.2007.00077.x PMC3823250

[35] Ramos JL, Sol Cuenca M, Molina-Santiago C, Segura A, Duque E, Gómez-García MR, et al Mechanisms of solvent resistance mediated by interplay of cellular factors in *Pseudomonas putida* . FEMS Microbiol Rev. 2015; 10.1093/femsre/fuv006 25934123

[36] Hallsworth JE, Prior BA, Nomura Y, Iwahara M, Timmis KN. Compatible solutes protect against chaotrope (ethanol)-induced, nonosmotic water stress. Appl Environ Microbiol. 2003;69: 7032–7034. 14660346 10.1128/AEM.69.12.7032-7034.2003 PMC309872

[37] Cray JA, Russell JT, Timson DJ, Singhal RS, Hallsworth JE. A universal measure of chaotropicity and kosmotropicity. Environ Microbiol. 2013;15: 287–296. 23145833 10.1111/1462-2920.12018

[38] Cray JA, Stevenson A, Ball P, Bankar SB, Eleutherio ECA, Ezeji TC, et al Chaotropicity: a key factor in product tolerance of biofuel-producing microorganisms. Curr Opinion Biotechnol. 2015;33: 228–259. 10.1016/j.copbio.2015.02.010 25841213

[39] Roy A, Kucukural A, Zhang Y. I-TASSER: a unified platform for automated protein structure and function prediction. Nat Protoc. 2010;5: 725–738. 10.1038/nprot.2010.5 20360767 PMC2849174

[40] Hall DR, Gourley DG, Leonard GA, Duke EM, Anderson LA, Boxer DH, et al The high-resolution crystal structure of the molybdate-dependent transcriptional regulator (ModE) from *Escherichia coli*: a novel combination of domain folds. Embo J. 1990;18: 1435–1446. 10.1093/emboj/18.6.1435 PMC1171233 10075916

