# Supplementary figures and images for: Dynamic Response of Pseudomonas putida S12 to Sudden Addition of Toluene and the Potential Role of the Solvent Tolerance Gene trgI
Source: PLoS One. 2015 Jul 16;10(7):e0132416. doi: 10.1371/journal.pone.0132416 (PMC4504468; doi:10.1371/journal.pone.0132416)

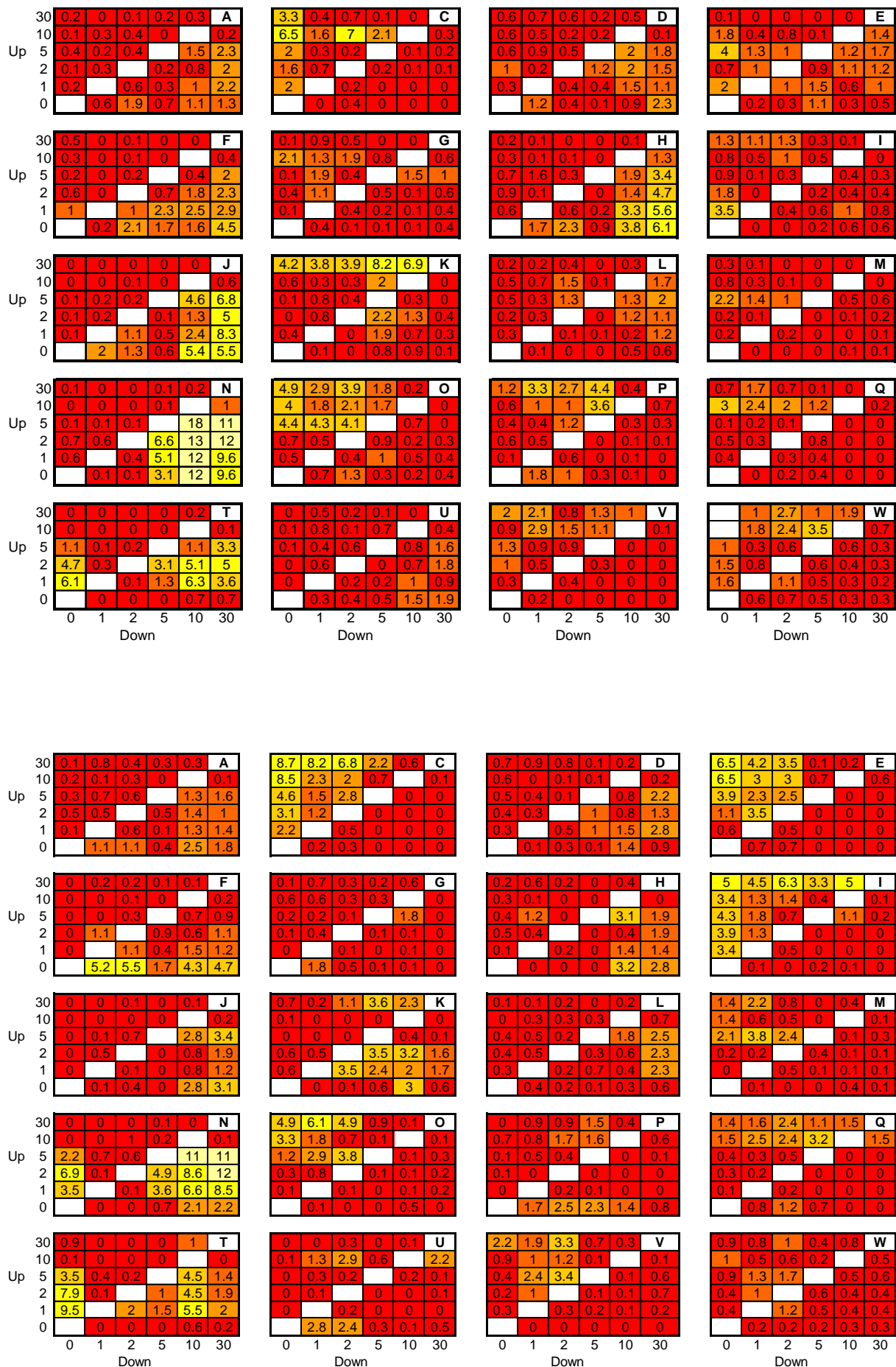

Supplement: S1 Fig — Values are –log(p-value). Values above the diagonal represent significance of overrepresentation of up-regulated genes and below the diagonal down-regulated genes are represented. Genes without COG (43 genes) and genes belonging to COG’s S (Function unknown, 2292 genes) and R (General function prediction only, 596 genes) are not shown. Abbreviations (with total amount of genes in brackets): A RNA processing and modification (16), C Energy production and conversion (260), D Cell division and chromosome partitioning (24), E Amino acid transport and metabolism (431), F Nucleotide transport and metabolism (90), G Carbohydrate transport and metabolism (164), H Coenzyme metabolism (131), I Lipid metabolism (166), J Translation, ribosomal structure and biogenesis (78), K Transcription (418), L DNA replication, recombination and repair (225), M Cell envelope biogenesis and outer membrane (214), N Cell motility (72), O Posttranslational modification, protein turnover and chaperones (117), P Inorganic ion transport and metabolism (246), Q Secondary metabolites biosynthesis, transport and catabolism (123), T Signal transduction mechanisms (239), U Intracellular trafficking and secretion (56), V Defence mechanisms (159), W Extracellular structure (4). (PDF) [file pone.0132416.s001.pdf]

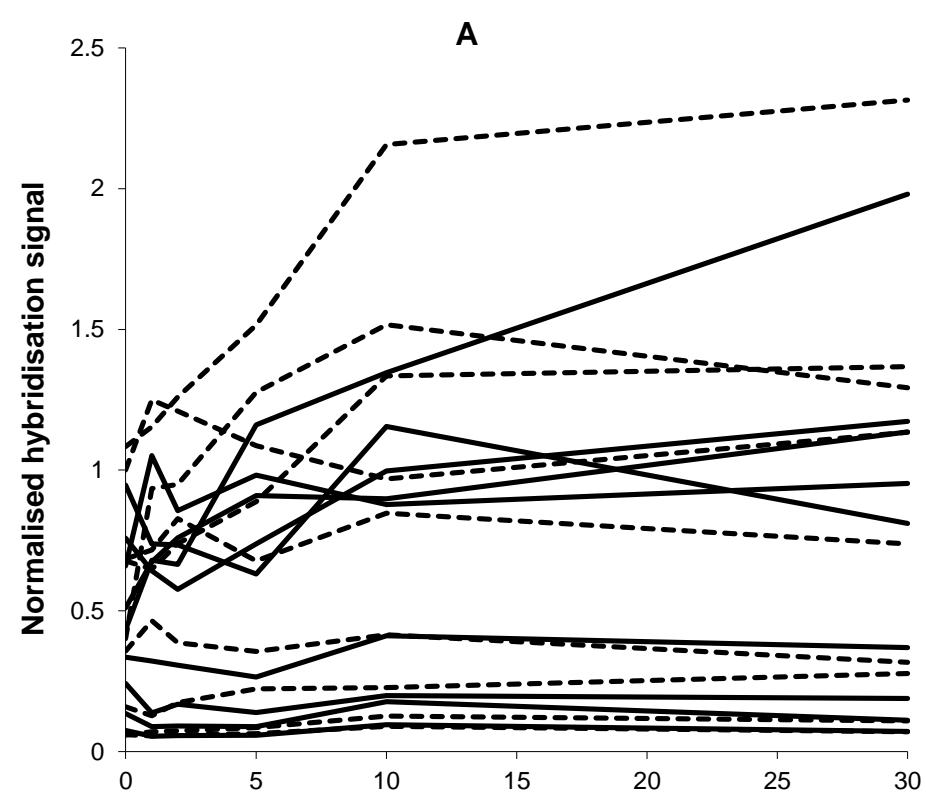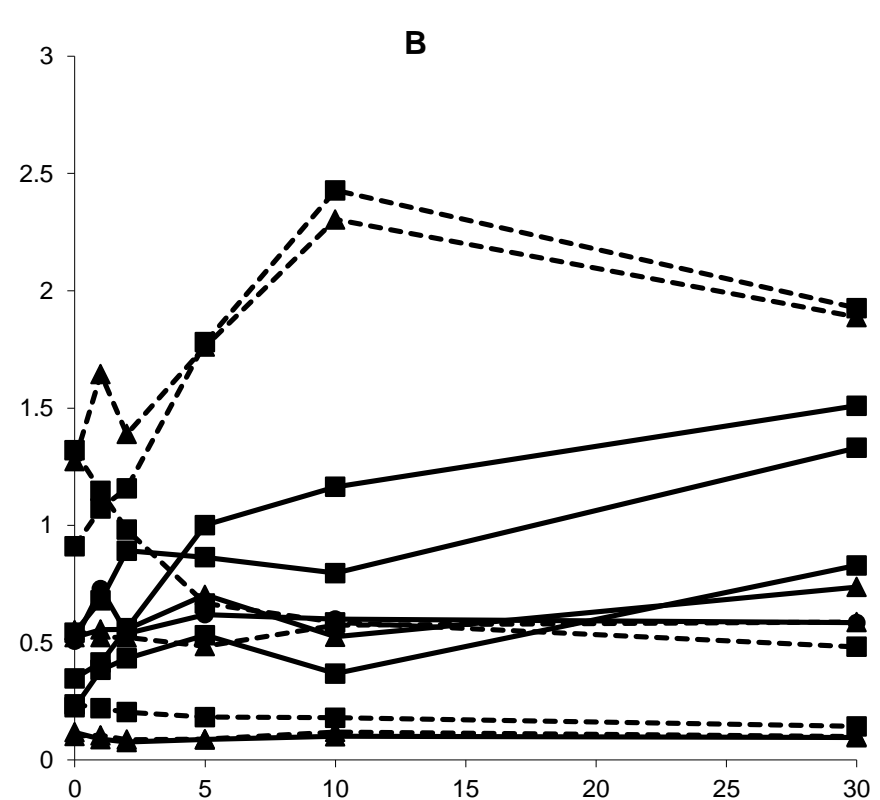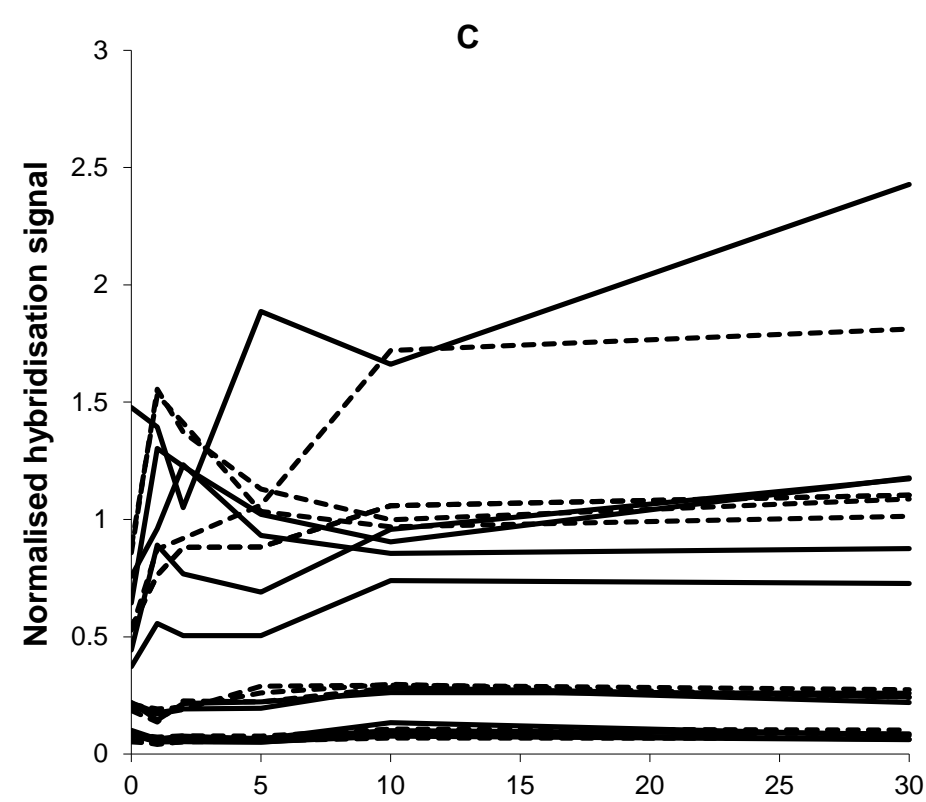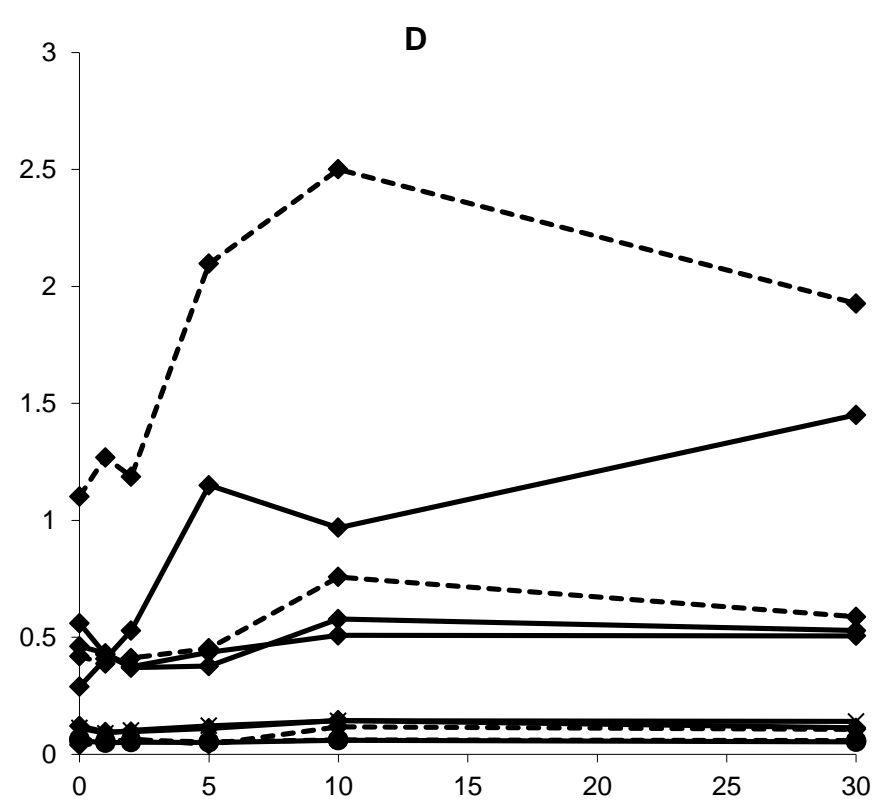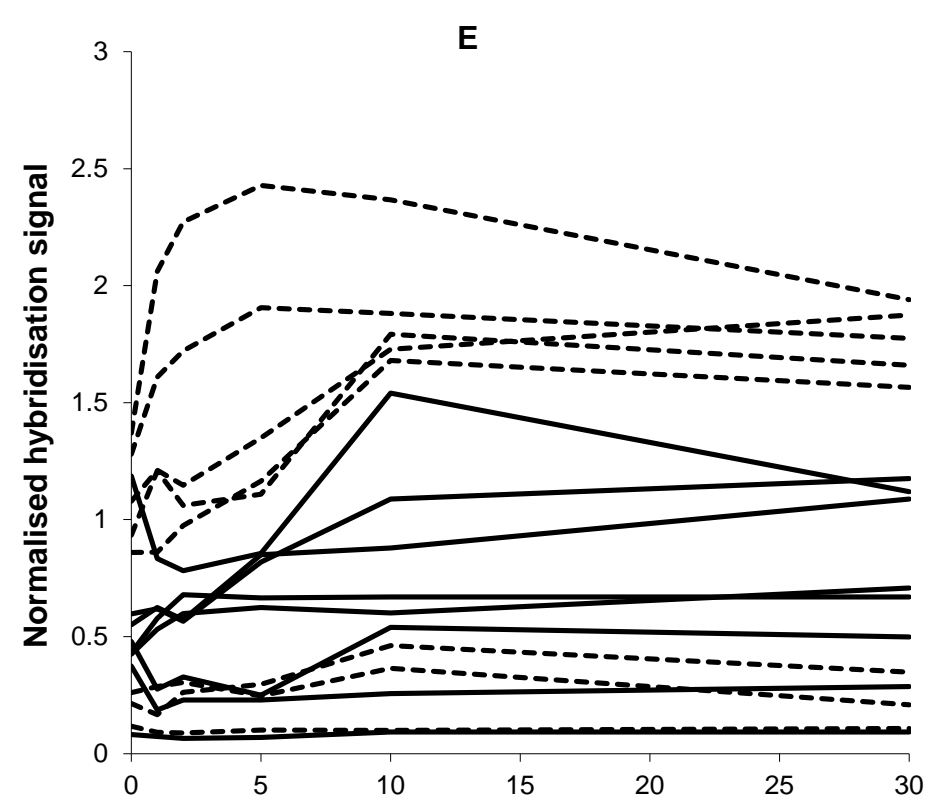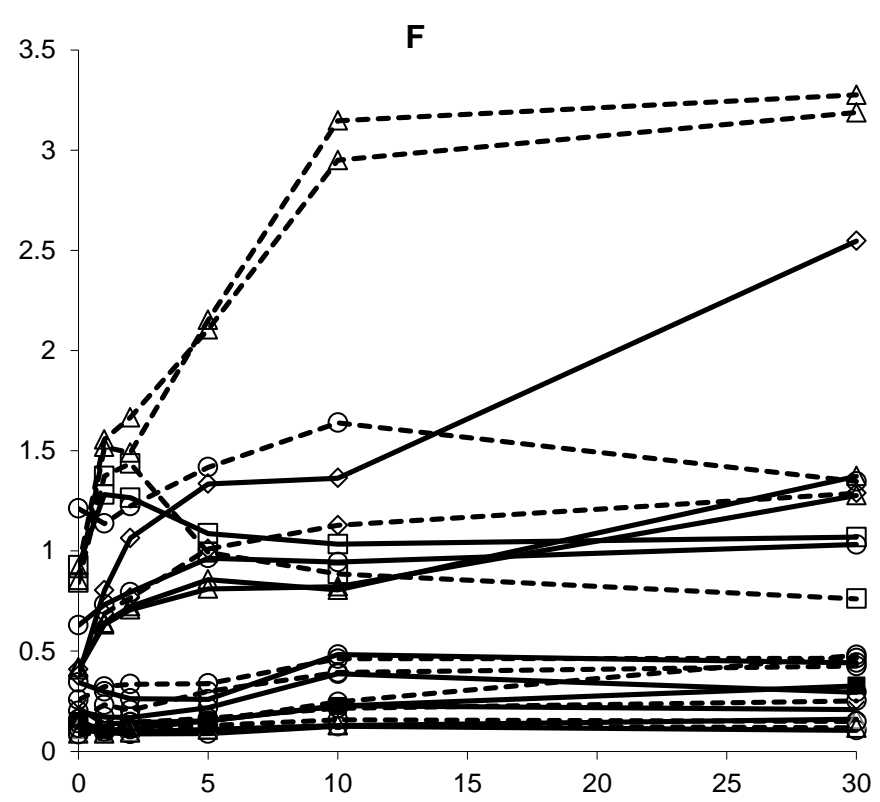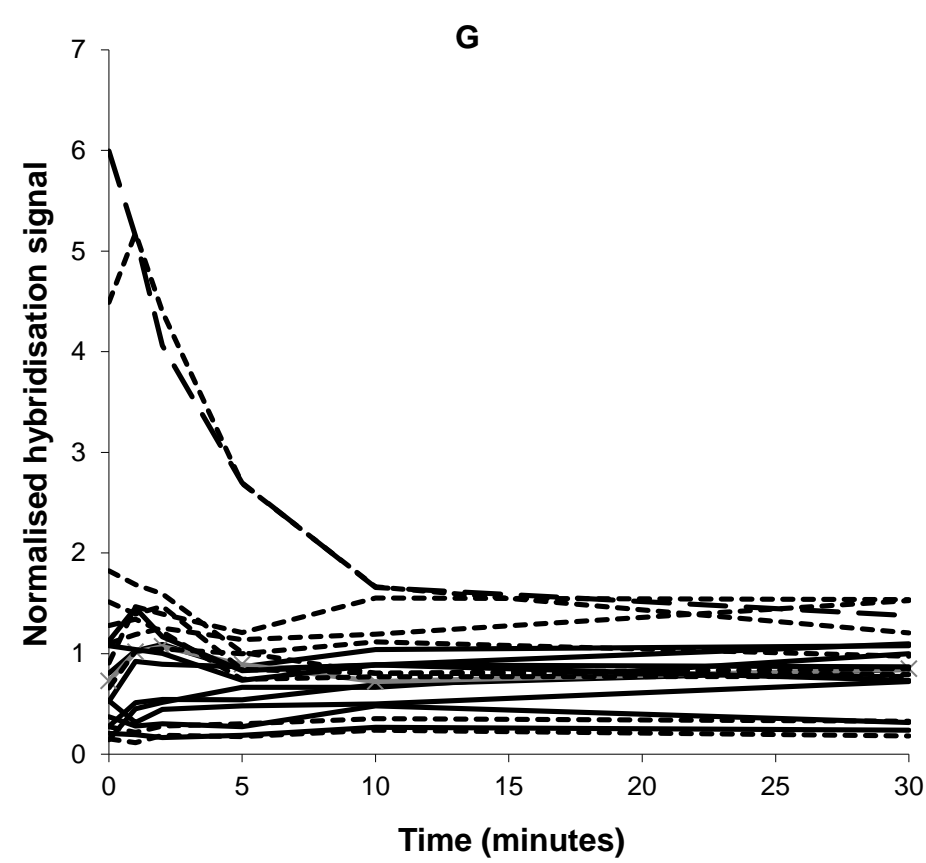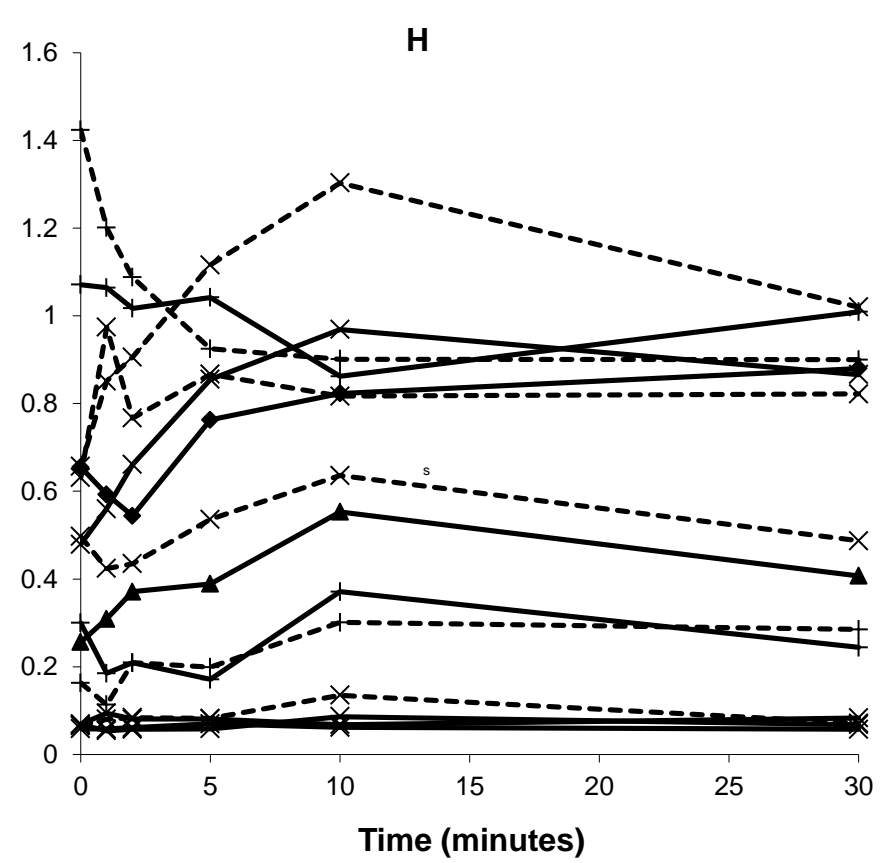

Supplement: S2 Fig — Solid lines: P. putida S12; Dotted lines P. putida S12ΔtrgI. (A) 3-ketoacyl-CoA thiolase; (B) ■ acetyl-CoA acetyltransferase and ▲ acyl-CoA dehydrogenase (1.3.99.-); (C) acyl-CoA dehydrogenase (EC 1.3.99.3); (D) ♦ acyl-CoA dehydrogenase, short-chain specific and ● alcohol dehydrogenase; (E) aldehyde dehydrogenase; (F) Δ enoyl-CoA hydratase/delta(3)-cis-delta(2)-trans-enoyl-CoA isomerase/3-hydroxyacyl-CoA dehydrogenase/3-hydroxybutyryl-CoA epimerase, ○ enoyl-CoA hydratase, ◊ glutaryl-CoA dehydrogenase and □ glutarate-CoA ligase; (G) long chain fatty acid-CoA ligase; (H) × membrane-bound aldehyde dehydrogenase iron-sulfur protein and + rubredoxin-NAD(+) reductase. (PDF) [file pone.0132416.s002.pdf]

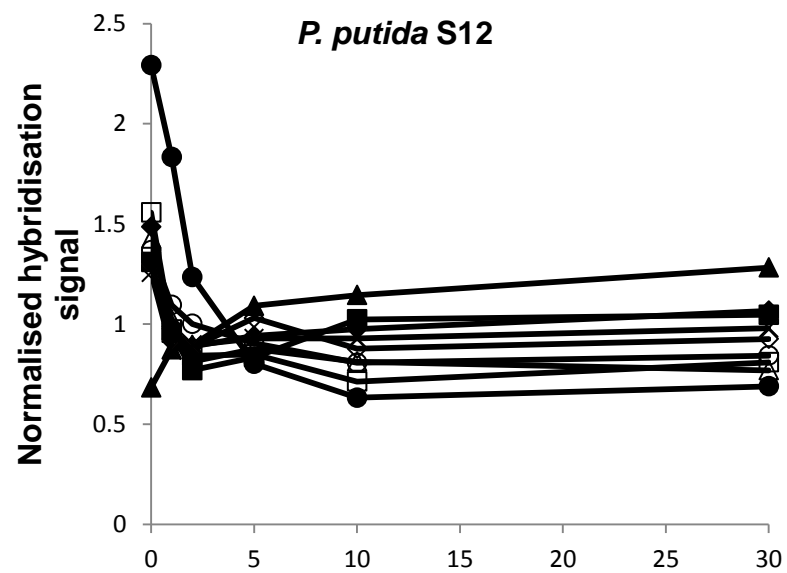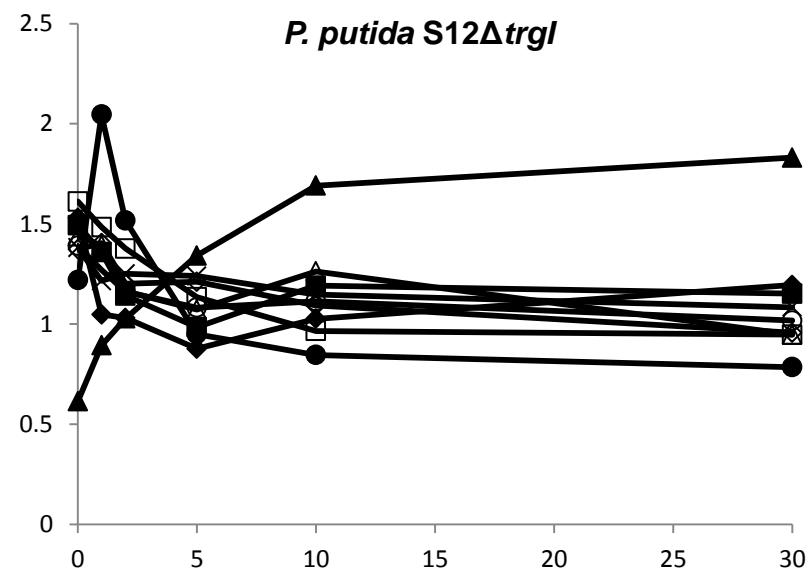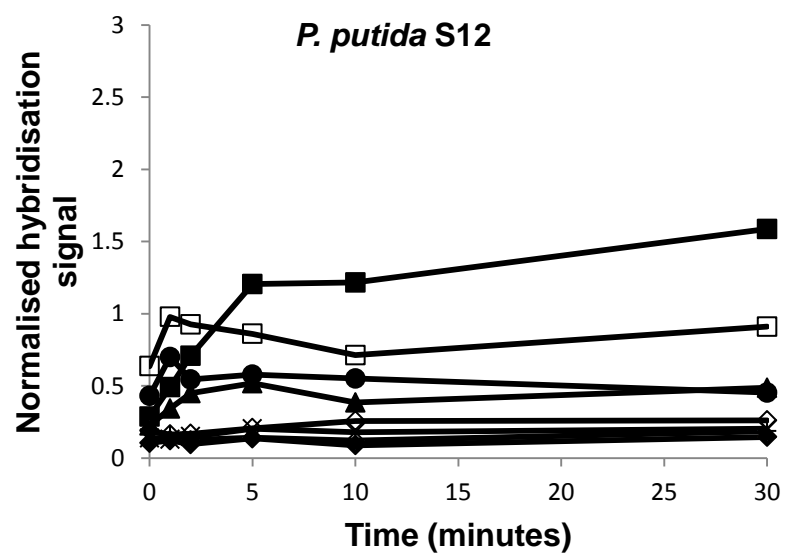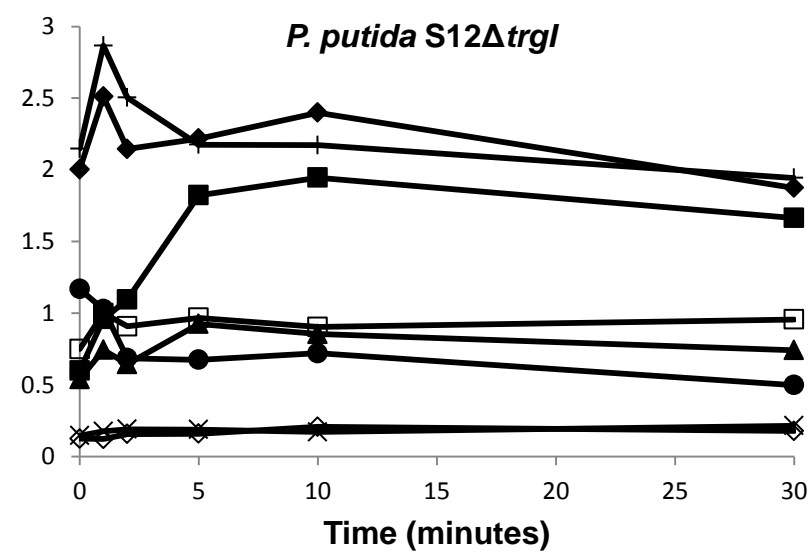

Supplement: S3 Fig — Upper panels: ♦ fabH (probe PS1201104_at), ■ fabH (probe PS1201105_at), ▲ fabH (probe PS1209460_at), ◊ fabD, ● biotin carboxylase (probe PS1206980_at), - biotin carboxylase (probe PS1202405_at), ∆ Biotin carboxyl carrier protein of acetyl-CoA carboxylase, × Acetyl-coenzyme A carboxylase carboxyl transferase subunit alpha, □ Acetyl-coenzyme A carboxylase carboxyl transferase subunit beta; Lower panels: ■ fabG (probe PS1201047_at), ● fabG (probe PS1204926_at), ▲ fabG (probe PS1201366_at), ♦ fabG (probe PS1200008_at), □ fabG (probe PS1204304_at), × fabG (probe PS1203087_at), + fabB (probe PS1200004_at), ◊ fabB (probe PS1201484_at). (PDF) [file pone.0132416.s003.pdf]

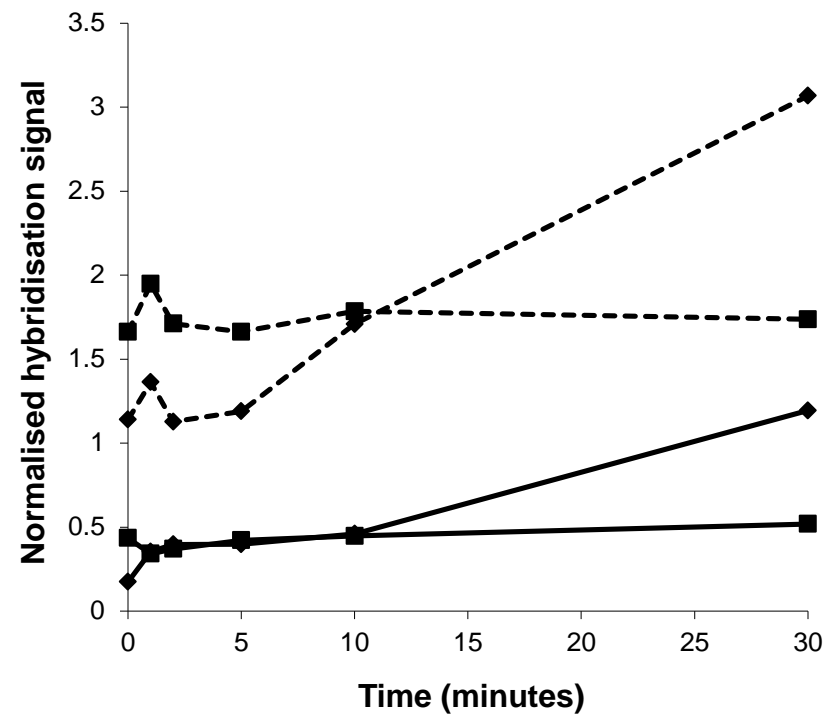

Supplement: S4 Fig — Solid lines: P. putida S12, dotted lines: P. putida S12ΔtrgI. ■ malate synthase, ♦ isocitrate lyase. (PDF) [file pone.0132416.s004.pdf]

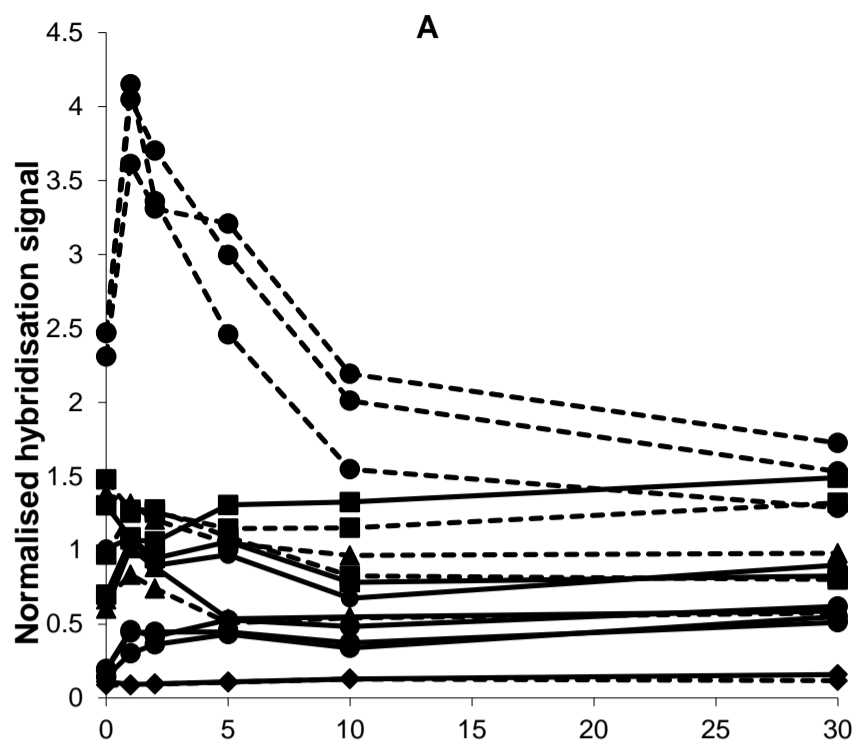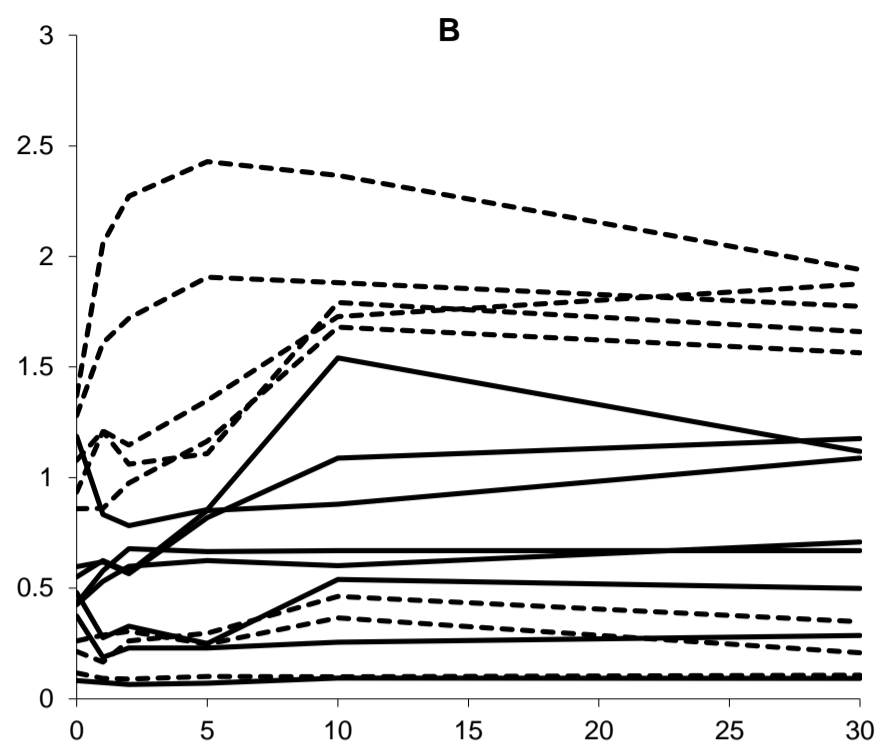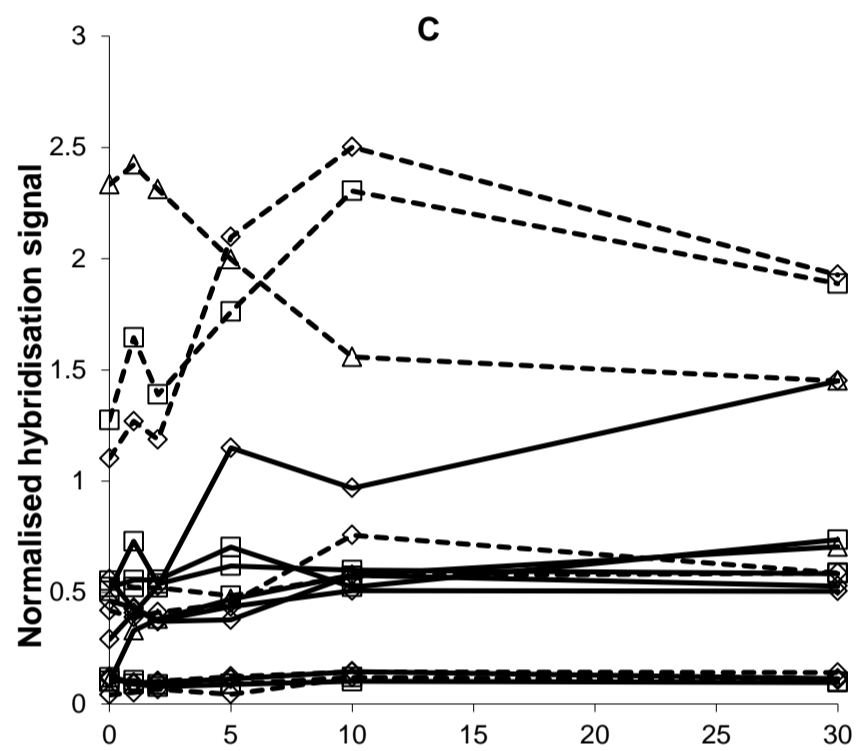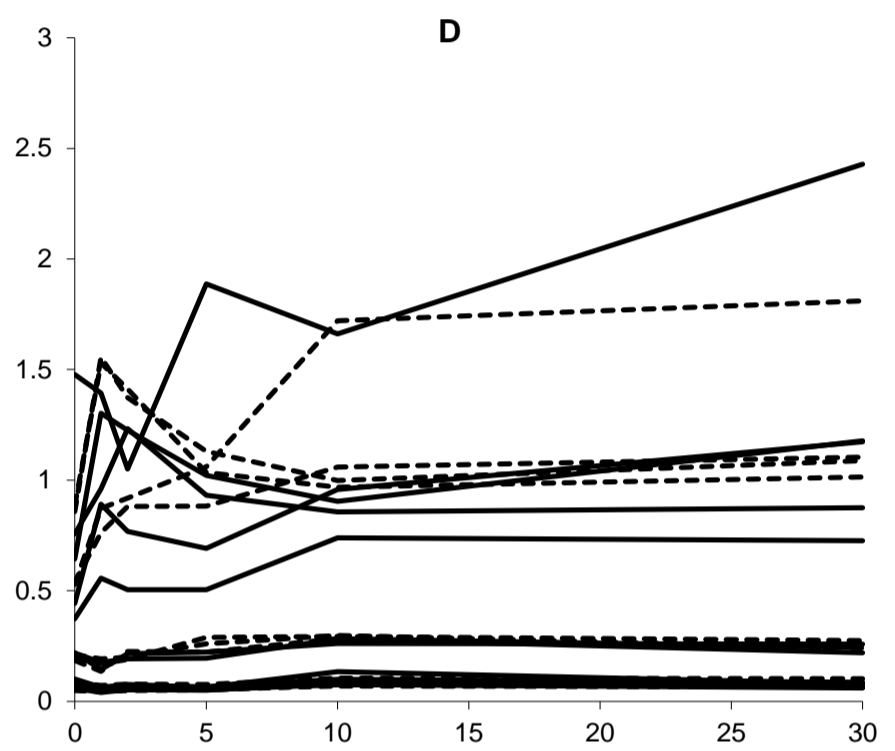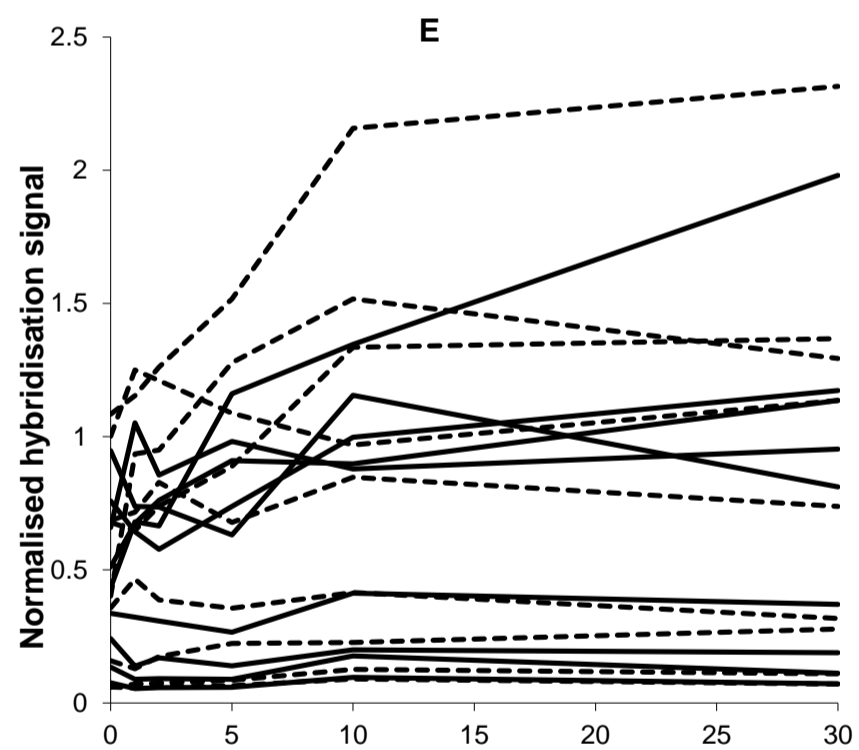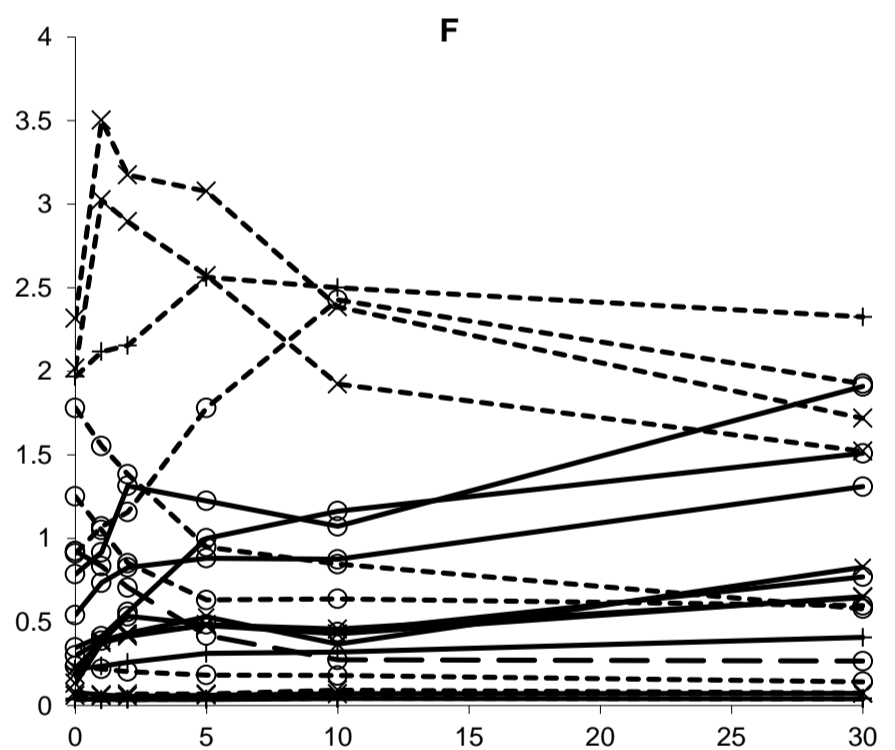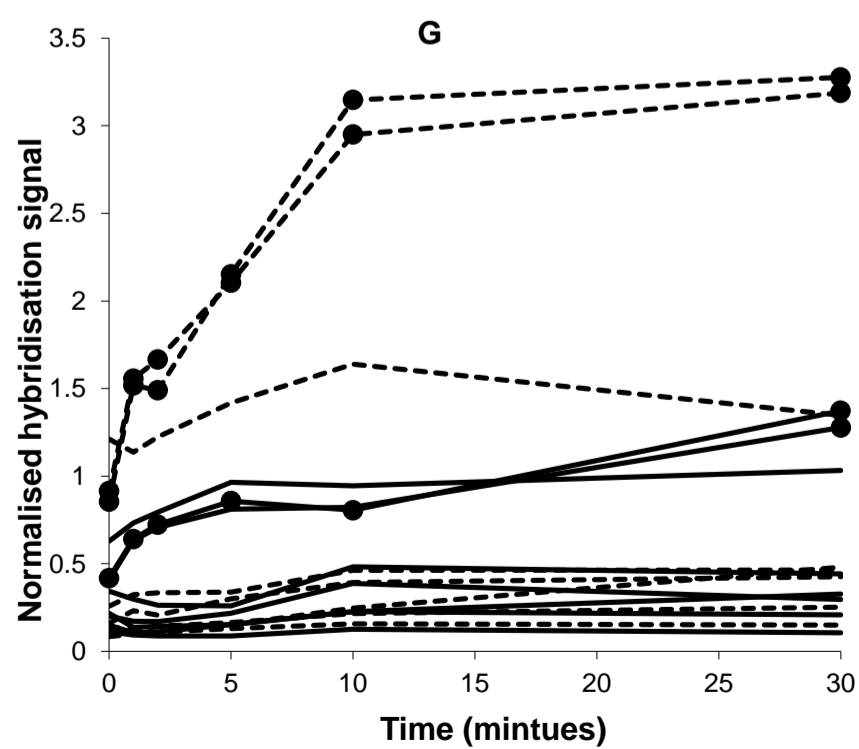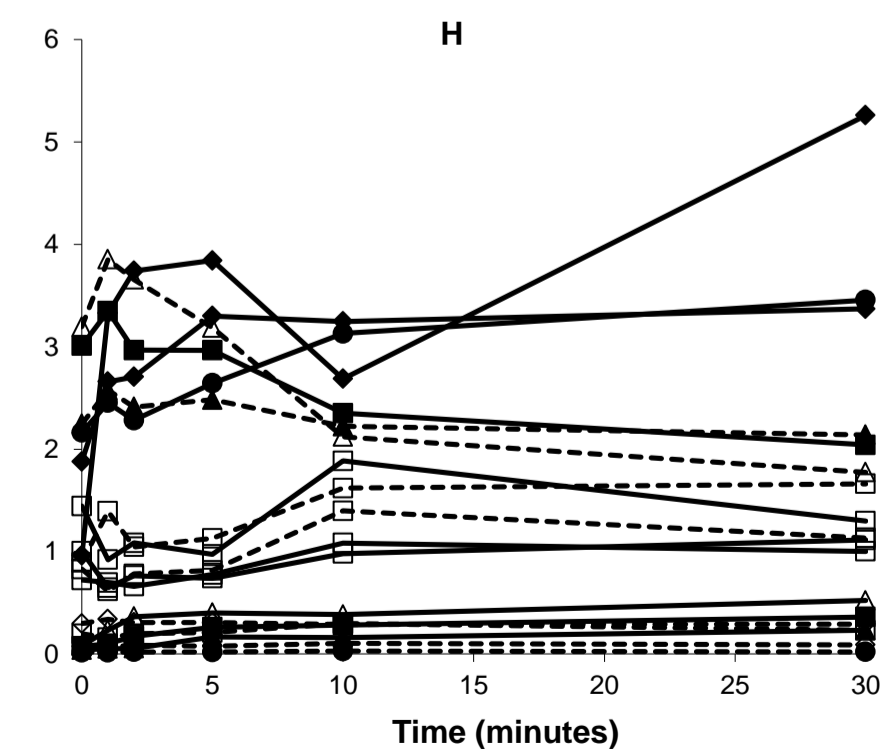

Supplement: S5 Fig — Solid lines: P. putida S12; Dotted lines P. putida S12ΔtrgI. (A) ■ 3-hydroxyisobutyrate dehydrogenase, ▲ leucine dehydrogenase, ♦ 3-hydroxyacyl CoA dehydrogenase, ● 2-oxoisovalerate dehydrogenase alpha or beta subunit; (B) aldehyde dehydrogenase; (C) Δ isovaleryl-CoA dehydrogenase, ◊ acyl-CoA dehydrogenase, short-chain specific, □ acyl-CoA dehydrogenase (EC 1.3.99.-); (D) acyl-CoA dehydrogenase (EC 1.3.99.3); (E) 3-ketoacyl-CoA thiolase; (F) × omega-amino acid—pyruvate aminotransferase, + dihydrolipoamide dehydrogenase, ○ acetyl-CoA acetyltransferase; (G) enoyl-CoA hydratase, ● enoyl-CoA hydratase / delta(3)-cis-delta(2)-trans-enoyl-CoA isomerase / 3-hydroxyacyl-CoA dehydrogenase / 3-hydroxybutyryl-CoA epimerase; (H) (Grey symbols) ● branched-chain amino acid aminotransferase, Δ methylcrotonyl-CoA carboxylase carboxyl transferase subunit, □ 3-hydroxyisobutyryl-CoA hydrolase, ◊ hydroxymethylglutaryl-CoA lyase, ♦ succinyl-CoA:3-ketoacid-coenzyme A transferase subunit A, ▲ methylcrotonyl-CoA carboxylase biotin-containing subunit, ■ methylglutaconyl-CoA hydratase. (PDF) [file pone.0132416.s005.pdf]

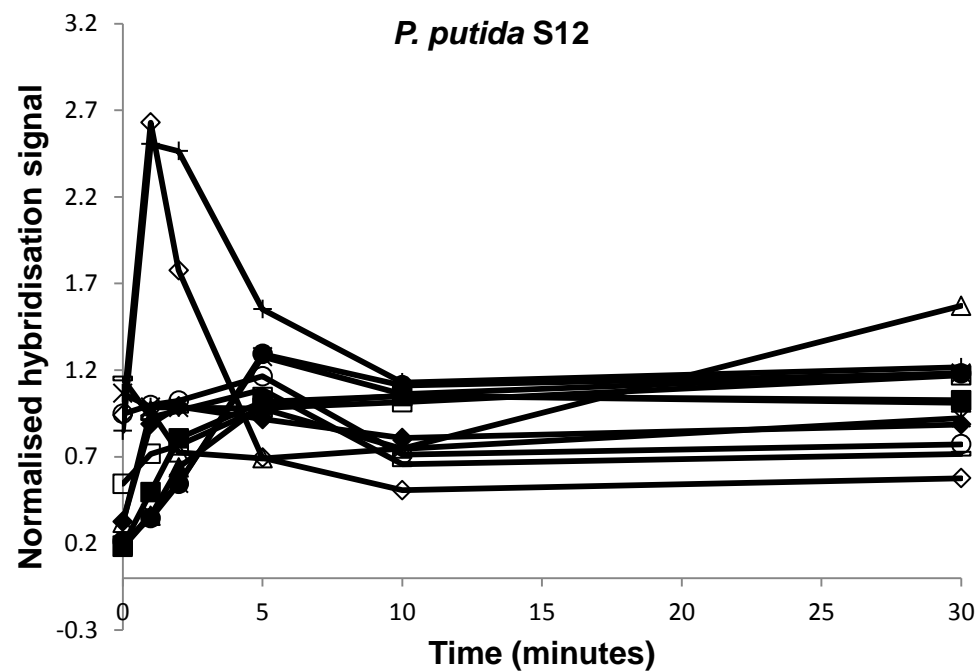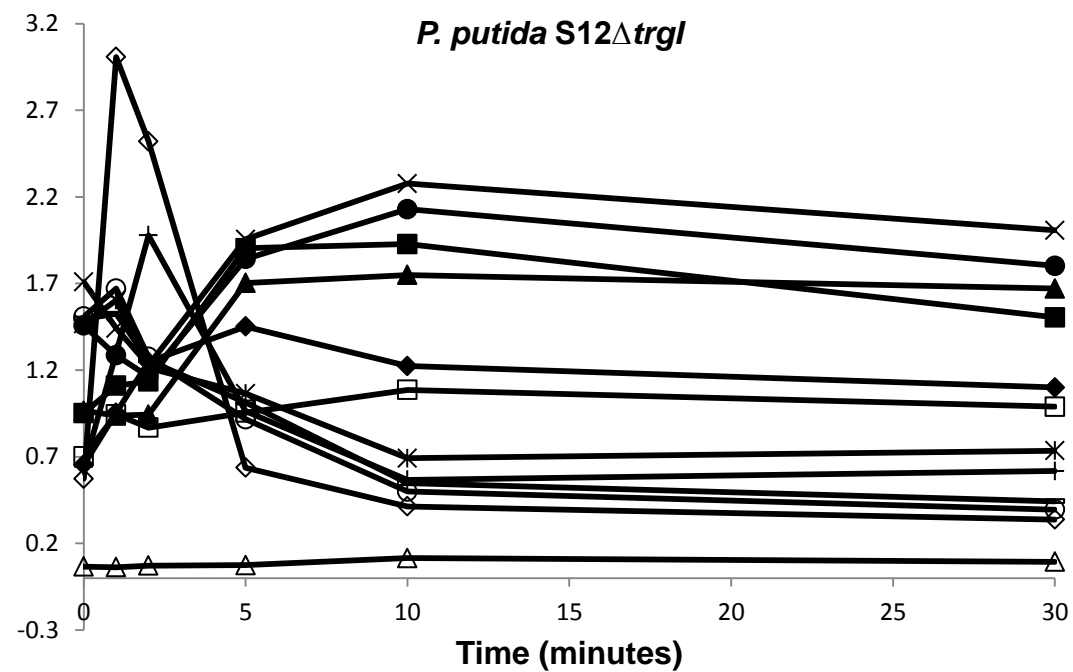

Supplement: S6 Fig — Black symbols: ♦ arginine deiminase, ▲ornithine carbamoyltransferase, ■ ornithine carbamoyltransferase, ● carbamate kinase, × carbamate kinase, ∆ ornithine cyclodeaminase, □ ornithine cyclodeaminase family protein; Grey symbols: ▲ aotM, × aotP, ● aotQ, ♦ arginine/ornithine antiporter, ■ arginine/ornithine antiporter. (PDF) [file pone.0132416.s006.pdf]

*P. putida* S12

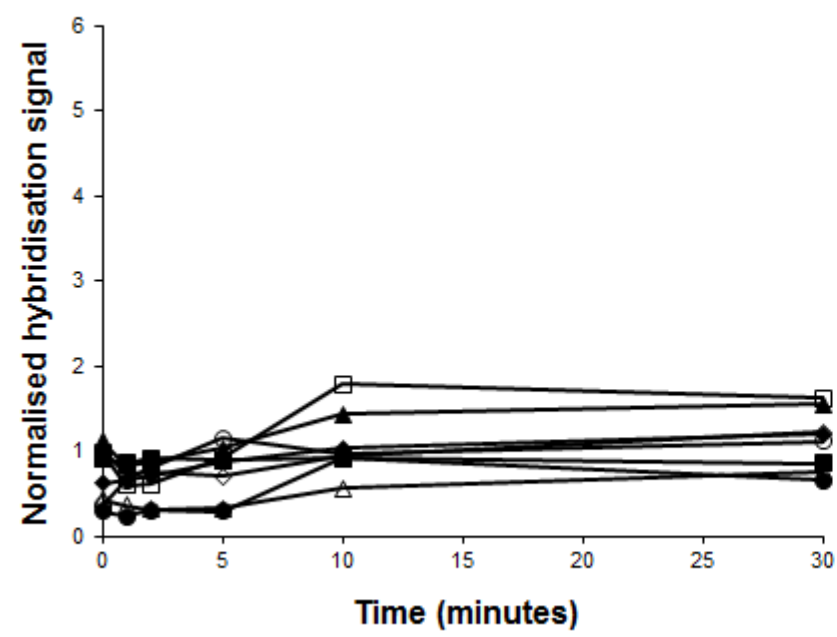

*P. putida* S12 $\Delta$ Trgl

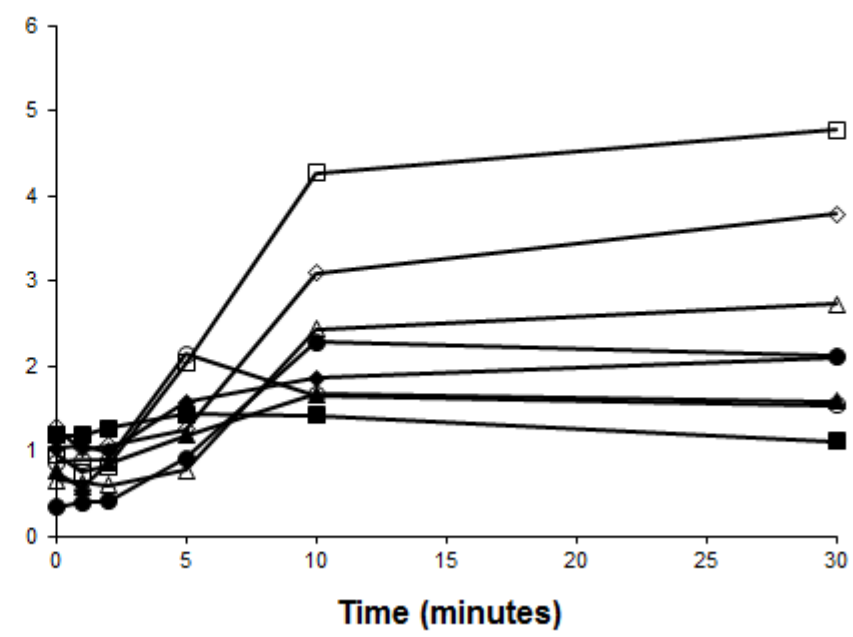

Supplement: S7 Fig — Black symbols: ♦ dsbA, ▲dsbD, ■ dsbC, ○ RPPX01161 tlpA, ● RPPX04273 tlpA; grey symbols: ♦ RPPX05428 dsbG, ▲ RPPX04274 dsbG, ■ dsbD. (PDF) [file pone.0132416.s007.pdf]

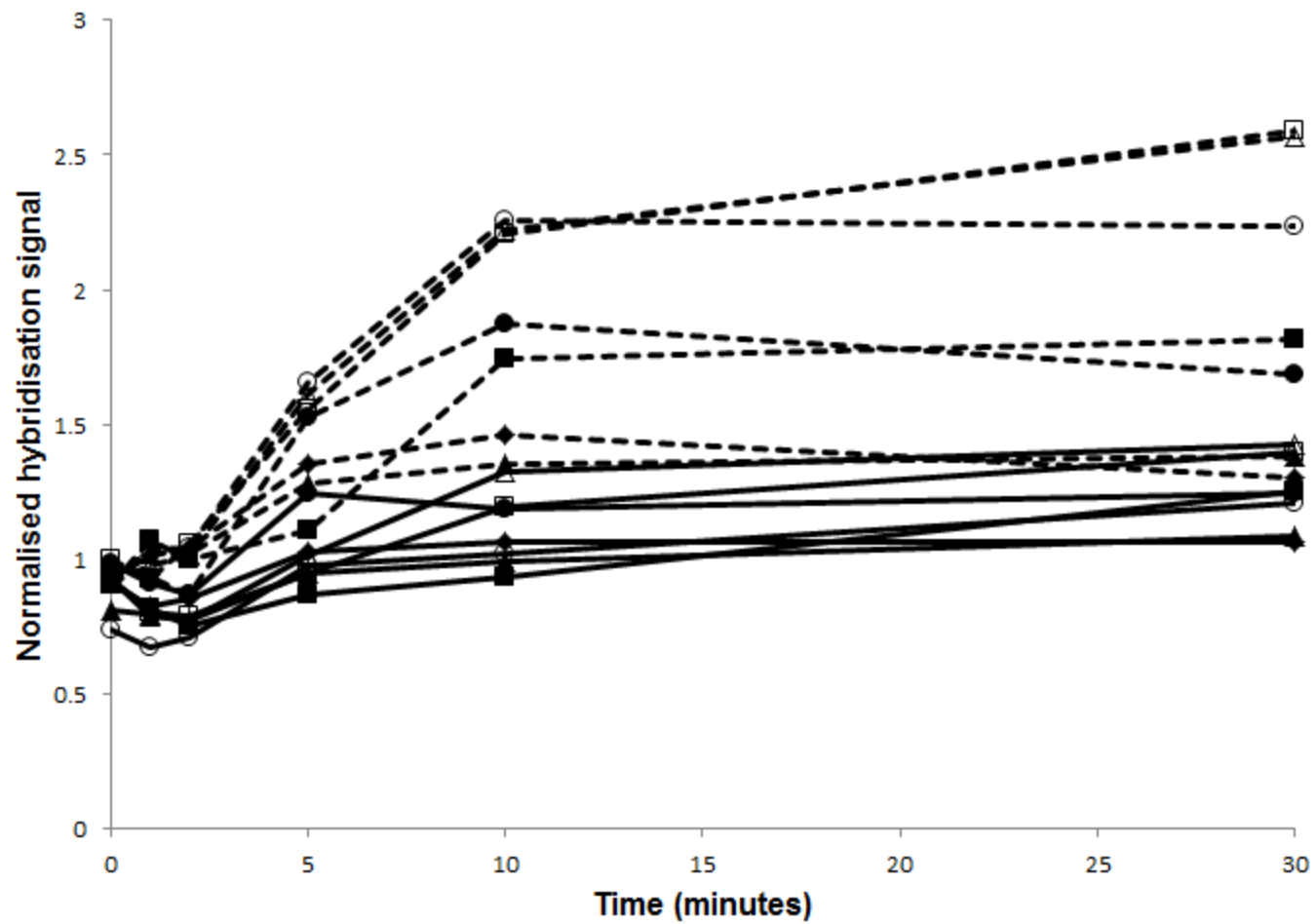

Supplement: S8 Fig — Solid lines: P. putida S12; Dotted lines P. putida S12ΔtrgI. ♦ groEL, ▲ groES, ● 33 kDa chaperonin (RPPX07096), ■ dnaJ, □ htpG (RPPX05623), ○ htpG (RPPX05624). (PDF) [file pone.0132416.s008.pdf]
